# Development and external validation of the eFalls tool: a multivariable prediction model for the risk of ED attendance or hospitalisation with a fall or fracture in older adults

**DOI:** 10.1093/ageing/afae057

**Published:** 2024-03-22

**Authors:** Lucinda Archer, Samuel D Relton, Ashley Akbari, Kate Best, Milica Bucknall, Simon Conroy, Miriam Hattle, Joe Hollinghurst, Sara Humphrey, Ronan A Lyons, Suzanne Richards, Kate Walters, Robert West, Danielle van der Windt, Richard D Riley, Andrew Clegg, The eFI+ investigators

**Affiliations:** Institute for Applied Health Research, University of Birmingham, Birmingham, UK; National Institute for Health and Care Research (NIHR) Birmingham Biomedical Research Centre, University of Birmingham, Birmingham, UK; Leeds Institute of Health Sciences, University of Leeds, Leeds, UK; Population Data Science, Swansea University Medical School, Swansea University, Swansea, UK; Academic Unit for Ageing and Stroke Research, University of Leeds, Bradford Teaching Hospitals NHS Foundation Trust, Bradford, UK; School of Medicine, Keele University, Keele, UK; Institute of Cardiovascular Science, University College London, London, UK; Institute for Applied Health Research, University of Birmingham, Birmingham, UK; National Institute for Health and Care Research (NIHR) Birmingham Biomedical Research Centre, University of Birmingham, Birmingham, UK; Population Data Science, Swansea University Medical School, Swansea University, Swansea, UK; Bradford District and Craven Health and Care Partnership, Bradford, UK; Population Data Science, Swansea University Medical School, Swansea University, Swansea, UK; Leeds Institute of Health Sciences, University of Leeds, Leeds, UK; Primary Care and Population Health, University College London, London, UK; Leeds Institute of Health Sciences, University of Leeds, Leeds, UK; School of Medicine, Keele University, Keele, UK; Institute for Applied Health Research, University of Birmingham, Birmingham, UK; National Institute for Health and Care Research (NIHR) Birmingham Biomedical Research Centre, University of Birmingham, Birmingham, UK; Academic Unit for Ageing and Stroke Research, University of Leeds, Bradford Teaching Hospitals NHS Foundation Trust, Bradford, UK

**Keywords:** falls, prediction model, prognosis, proactive, prevention, older people

## Abstract

**Background:**

Falls are common in older adults and can devastate personal independence through injury such as fracture and fear of future falls. Methods to identify people for falls prevention interventions are currently limited, with high risks of bias in published prediction models. We have developed and externally validated the eFalls prediction model using routinely collected primary care electronic health records (EHR) to predict risk of emergency department attendance/hospitalisation with fall or fracture within 1 year.

**Methods:**

Data comprised two independent, retrospective cohorts of adults aged ≥65 years: the population of Wales, from the Secure Anonymised Information Linkage Databank (model development); the population of Bradford and Airedale, England, from Connected Bradford (external validation). Predictors included electronic frailty index components, supplemented with variables informed by literature reviews and clinical expertise. Fall/fracture risk was modelled using multivariable logistic regression with a Least Absolute Shrinkage and Selection Operator penalty. Predictive performance was assessed through calibration, discrimination and clinical utility. Apparent, internal–external cross-validation and external validation performance were assessed across general practices and in clinically relevant subgroups.

**Results:**

The model’s discrimination performance (c-statistic) was 0.72 (95% confidence interval, CI: 0.68 to 0.76) on internal–external cross-validation and 0.82 (95% CI: 0.80 to 0.83) on external validation. Calibration was variable across practices, with some over-prediction in the validation population (calibration-in-the-large, −0.87; 95% CI: −0.96 to −0.78). Clinical utility on external validation was improved after recalibration.

**Conclusion:**

The eFalls prediction model shows good performance and could support proactive stratification for falls prevention services if appropriately embedded into primary care EHR systems.

## Key Points

- Falls can devastate personal independence through injury such as fracture, and fear of future falls.
- Proactive falls prevention could help prevent both future injury and falls, but existing prediction models have limitations.
- We have developed and externally validated the eFalls prediction model to support proactive falls prevention services.
- eFalls has good predictive performance and is suitable for integrating into primary care electronic health record systems.
- eFalls could help transform how falls prevention services are delivered in the UK and, in the future, internationally.

## Introduction

Falls are common in older age, with around one-third of people aged >65 experiencing at least one fall each year [1]. Falls have a potentially devastating impact on independence through associated injuries such as fractures, and decreased ability to carry out activities of daily living [2, 3]. The fear of experiencing future falls can be hugely constraining, affecting around half of those who have fallen previously, with resulting activity avoidance and social isolation [4–6]. Falls also have a major impact on health and care systems globally [4], as a leading cause of hospitalisation in older people [7]. The incidence of falls is also projected to rise in line with the global ageing demographic [8].

Falls typically result from interaction between a range of factors, for example, gait and balance impairment, sensory impairment, medications and environmental factors [2]. Evidence indicates that major reductions in falls risk can be achieved through interventions targeting these risk factors, including resistance exercise training, or through multifactorial assessment and treatment [9].

Multicomponent falls prevention interventions are supported as clinically and cost-effective interventions in UK and international guidelines [10, 11]. The 2022 World Falls Prevention Guidelines recommend stratifying older people into levels of risk based on having fallen in the past 12 months, and, for those who have fallen, providing tailored interventions based on severity of the fall and whether gait and balance are impaired. This approach is largely reactive, whereby a history of falling is a pathway entry criterion, and could be considered a limitation. Proactive falls prevention for people who have not yet fallen but are at high risk could help prevent both future injury and fall-related fear. However, using performance-based tests to support patient stratification requires additional clinical resources, which is challenging in time-pressured environments and is a barrier to implementation [12]. The use of routinely collected electronic health records (EHR) to automate identification of falls risk has considerable potential to support more efficient implementation of falls prevention interventions.

A 2021 systematic review of prognostic models for falls in community-dwelling older adults identified 72 falls prediction models, only three of which were externally validated [13]. Discrimination (reported in only 40 cases) was wide ranging and at best moderate on external validation (where conducted). Only seven models reported calibration, which was moderate to poor. Many potentially important predictors were rarely considered in these existing models, such as visual impairment (7 models, 9.7% of those considered); dizziness, polypharmacy and body mass index (4 models each, 5.6%); or dementia diagnosis (2, 2.8%). Models were largely based on prospective cohort or survey data, did not adequately report their outcome definition and required additional clinical information to be gathered. All of these models were found to be at a high risk of bias, with concerns in the analysis domain for all studies, making them unsuitable for implementation in practice.

A 2022 EHR-based prediction model for 10-year falls risk was promising but was limited to patients with an indication for antihypertensive treatment [14], thus does not necessarily apply to the wider population at risk of falls. It had good discrimination on external validation, but over-predicted falls risk at 1 year (the usual time horizon for stratifying patients for falls prevention interventions). Furthermore, frailty was only considered only as a composite score, rather than as individual frailty components (for example, those from the electronic Frailty Index [eFI] [15]).

Thus, in this study we aimed to develop and externally validate a prediction model using routinely collected primary care data, to accurately predict the risk of emergency department (ED) attendance/hospitalisation with fall or fracture (as an indicator of a fall injury) within 1 year of assessment in general practice, for all individuals aged ≥65 years.

## Methods

Two retrospective cohorts were used in the development and external validation of the eFalls prediction model. Model development was conducted in the Secure Anonymised Information Linkage (SAIL) Databank, which contains longitudinal, routinely collected, anonymised EHR data sources from around 5 million people across Wales, with linked primary care, ED attendance, hospital admissions and Office for National Statistics mortality data [16]. SAIL uses Read version 2 clinical coding ontology in primary care data.

External validation was performed in Connected Bradford, which includes linked health and social care data from around 800,000 residents of Bradford and Airedale, located in the north of England [17]. The included data span five NHS Trusts, 86 general practices and linked health, education, social care, environmental and local government data. Connected Bradford uses Systematised Nomenclature of Medicine—Clinical Terms (SNOMED-CT) as the clinical coding ontology in primary care.

Both included data sources have been described in detail elsewhere [16, 17].

### Population

Model development was conducted in patients registered with a SAIL-providing general practice on 1 April 2018. External validation took place in those registered with a Connected Bradford general practice on 1 January 2019. Eligible patients were defined the same way in both populations as those with linked data, aged ≥65 years.

### Outcomes

The outcome was any (one or more) ED attendance or hospital admission for a fall or fracture (as an indicator of an injurious fall) within 12 months of their baseline predictor assessment. Outcomes were identified in SAIL through linkage with the Emergency Department Dataset and Patient Episode Database for Wales and in Connected Bradford through linked secondary care data.

A list of ICD-10 codes used to define fall/fractures is included in Supplementary Table S2.3.

### Candidate predictors

Frailty is a recognised predictor of falls, regardless of which frailty model is used, but there is currently uncertainty regarding which individual frailty components contribute to falls risk [18]. The electronic Frailty Index (eFI), which has been externally validated for a range of outcomes and has good convergent validity [15, 19], has been shown to identify older people at increasing risk of falling [20].

The eFI includes components only infrequently considered in other frailty measures (e.g. dementia, activities of daily living (ADL) impairment, visual impairment, polypharmacy), though it is uncertain which (if any) of the eFI components are themselves associated with falls risk, nor how strong individual associations are. Thus, candidate predictors in the eFalls model included the 36 components of the eFI [15], supplemented with variables available within routinely collected primary care data. These 44 additional variables were informed by a systematic review, funded by the National Institute for Health Research School for Primary Care Research (NIHR-SPCR) Evidence Synthesis Working Group [21], and targeted scoping reviews.

Candidate predictor variables were constructed by organising individual EHR SNOMED-CT codes into groups, with back transformation to Read version 2 using NHS England Technology Reference Update Distribution lists, with clinical validation of all new predictor variables in both SNOMED-CT and Read version 2.

### Sample size

Using recommendations to minimise overfitting and estimate risks precisely [22], the minimal sample size required for model development was 50,927 with 2,445 events, based on an anticipated 90 predictor parameters. The number of fall/fractures in SAIL far exceeded this at 32,097.

Assuming performance similar to that of internal validation, a minimum of 10,882 people (523 events) were required for external validation to achieve precise estimates of predictive performance [23]. Connected Bradford contained 81,685 participants with 2,389 events.

Further calculation details are given in Appendix S2.

### Statistical analysis

Model development and internal validation analyses were conducted in Stata version 17 (StataCorp). External validation analyses were conducted in R version 4.2.3. This report adheres to the TRIPOD-Cluster checklist for transparent reporting of multivariable prediction models developed or validated using clustered data [24].

### Missing data

Missing data were treated the same way in both model development and validation data. Where individual diagnoses or prescriptions were not recorded for a patient, they were assumed not to be present. Similarly, where a fall/fracture was not coded within 12 months, it was assumed that no fall/fracture occurred. For other predictors, this study employs missing indicators, with missing observations allocated to ‘missing’ groups for categorical variables [25], to be aligned with the approach intended at model implementation [26].

### Model development

Researchers at the University of Birmingham conducted the model development and internal validation. The predicted risk of a fall/fracture was modelled using multivariable logistic regression with a Least Absolute Shrinkage and Selection Operator (LASSO) penalty. Clustering of participants by general practice was not accounted for at model development, but predictive performance was assessed by practice. The LASSO tuning parameter, lambda, was chosen to minimise the cross-validation function on 10-fold cross-validation. Continuous predictors (age and polypharmacy) were modelled using second-order fractional polynomials, with functional forms chosen in the presence of all predictors. Transformed age and polypharmacy terms were then used as candidate predictors in the LASSO regression.

### Internal validation

Internal validation was conducted using bootstrapping with 25 samples (chosen for computational efficiency due to the use of big data), sampling with replacement from the model development population. The predictive performance of the model developed in each bootstrap was assessed in both that sample and the original data, to gain estimates of optimism, and model performance estimates were adjusted accordingly. Model stability was assessed through probability distribution and calibration instability plots [27].

Performance was assessed through calibration, discrimination and clinical utility. Calibration was quantified using the calibration slope, calibration-in-the-large (CITL), and the ratio of Observed to Expected outcomes (O/E). Calibration plots show the agreement between predicted and observed outcome probabilities within groups (defined by 20ths of outcome risk), and across all individuals using smooth (loess) calibration curves. Discrimination was assessed using the c-statistic. Clinical utility was quantified using net benefit and decision curve analysis [28, 29]. Risk thresholds of clinical interest for guiding decision making were specified a priori by a clinical user group. Thresholds between 10% and 25% were chosen, thus net benefit in this range was of most interest.

Variability in model performance was assessed across individual general practices [30], plotting performance estimates against their standard errors (practices with <10 events omitted from visualisations to preserve anonymity). Predictive performance was summarised across all practices, on appropriate scales [31], with random-effects meta-analysis estimated using restricted maximum likelihood. Confidence intervals for pooled estimates were derived using the Hartung–Knapp–Sidik–Jonkman variance correction [32].

### Internal–external cross-validation

An internal–external cross-validation approach was used for further validation in the model development dataset [33, 34], across subgroups by ranked Welsh Index of Multiple Deprivation (WIMD, 2019 version). In each cycle, the model development process (as described above) was repeated using all-but-one of the groups. This model was then applied to the omitted data, and its predictive performance was assessed. Following all cycles, performance estimates were summarised using a random-effects meta-analysis, as specified above.

### External validation

External validation was conducted by researchers at University of Leeds, independent to the model development team. The prediction model equation was applied to the external data to predict outcome risks for each participant in the dataset. Predictive performance was evaluated as described above.

Performance was also assessed in clinically relevant subgroups, with the above assessment repeated by sex, body mass index (BMI) category, Indices of Multiple Deprivation (IMD) subgroups (grouped at quintile values) and frailty group (fit, mild, moderate or severe [15]).

Given the importance of model calibration for the clinical application of clinical prediction models, where calibration was suboptimal in the new population, recalibration to the external data was considered [35]. Updating the intercept was used to account for differences in outcome frequency, while adjustment of all regression coefficients by the same adjustment factor accounted for any under- or overfitting at model development [36]. This was implemented by fitting a new logistic regression model in the external validation data, with the linear predictor value from the eFalls model as the only variable. Apparent performance of this recalibrated model was assessed in the Connected Bradford data, as described above.

### Patient and public involvement

Patient and public representatives were involved in the development of the research question, project implementation, setting risk thresholds for examination of net benefit and interpretation of findings. Use of the SAIL Databank was approved by an independent Information Review Governance Panel that contained members of the public.

## Results

### Summary of development and validation datasets

The model development data constituted a combination of eligible patients from 455 general practices across Wales. Of the 660,417 participants available for model development, 32,097 (4.9%) experienced a fall/fracture resulting in ED attendance or hospitalisation within 12 months. The external validation data, from Connected Bradford, contained 81,685 eligible participants, across 76 practices, with 2,389 (2.9%) fall/fracture events.

A comparison of population characteristics for model development and external validation cohorts is given in Table 1 and Supplementary Table S3.1.

**Table 1:** Descriptive statistics for model development and external validation cohorts, stratified by outcome status at 12 months

	Model development data			External validation data		
	Total	Fall/fracture	No fall/fracture	Total	Fall/fracture	No fall/fracture
*n*	*660,417*	*32,097 (4.9)*	*628,320 (95.1)*	*81,685*	*2,389 (2.9)*	*79,296 (97.1)*
Male, *n* (%)	311,742 (47.2)	11,423 (35.6)	300,319 (47.8)	37,319 (45.7)	872 (36.5)	36,447 (46.0)
Age (years), median [LQ to UQ]	73 [69 to 80]	79 [72 to 85]	73 [69 to 80]	74 [69 to 81]	83 [76 to 88]	74 [69 to 80]
Polypharmacy, median [LQ to UQ]	4 [0 to 9]	8 [4 to 13]	4 [0 to 9]	4 [1 to 7]	7 [4 to 11]	4 [1 to 7]
WIMD/IMD						
1. Most deprived	85,421 (12.9)	5,935 (18.5)	79,486 (12.7)	18,600 (22.8)	625 (26.2)	17,975 (22.7)
2. –	102,410 (15.5)	6,616 (20.6)	95,794 (15.3)	12,162 (14.9)	372 (15.6)	11,790 (14.9)
3. –	108,496 (16.4)	5,773 (18)	102,723 (16.4)	13,337 (16.3)	473 (19.8)	13,864 (17.5)
4. –	111,726 (16.9)	5,533 (17.2)	106,193 (16.9)	11,172 (13.7)	276 (11.6)	10,896 (13.7)
5. Least deprived	122,619 (18.6)	6,356 (19.8)	116,263 (18.5)	7,582 (9.3)	212 (8.9)	7,370 (9.3)
Missing	129,745 (19.7)	1,884 (5.9)	127,861 (20.4)	17,832 (21.8)	431 (18.0)	17,401 (21.9)
eFI score, median [LQ to UQ]	0.11 [0.06 to 0.19]	0.19 [0.11 to 0.28]	0.11 [0.03 to 0.19]	0.17 [0.08 to 0.25]	0.33 [0.22 to 0.42]	0.17 [0.08 to 0.25]
Frailty category*						
Fit	366,629 (55.5)	9,062 (28.2)	357,567 (56.9)	32,732 (40.0)	188 (7.9)	32,544 (41.0)
Mild	193,525 (29.3)	11,452 (35.7)	182,073 (29.0)	24,694 (30.2)	433 (18.1)	24,261 (30.6)
Moderate	77,792 (11.8)	8,083 (25.2)	69,709 (11.1)	14,496 (17.7)	747 (31.3)	13,749 (17.3)
Severe	22,471 (3.4)	3,500 (10.9)	18,971 (3.0)	9,763 (12.0)	1,021 (42.7)	8,742 (11.0)
BMI category						
Underweight	12,642 (1.9)	1,750 (5.5)	10,892 (1.7)	1,742 (2.1)	123 (5.1)	1,619 (2.0)
Normal	121,946 (18.5)	9,286 (28.9)	112,660 (17.9)	24,862 (30.4)	890 (37.3)	23,972 (30.2)
Overweight	158,676 (24)	8,177 (25.5)	150,499 (24)	29,631 (36.3)	748 (31.3)	28,883 (36.4)
Obese	136,646 (20.7)	6,788 (21.2)	129,858 (20.7)	21,698 (26.6)	501 (21.0)	21,197 (26.7)
Missing	230,507 (34.9)	6,096 (19)	224,411 (35.7)	3,752 (4.6)	127 (5.3)	3,625 (4.6)
Smoking						
Never	302,363 (45.8)	13,318 (41.5)	289,045 (46)	59,295 (72.6)	1,717 (71.9)	57,562 (72.6)
Ex	271,248 (41.1)	14,756 (46)	256,492 (40.8)	16 (0.0)	1 (0.0)	15 (0.0)
Current	86,806 (13.1)	4,023 (12.5)	82,783 (13.2)	22,390 (27.4)	671 (28.1)	21,719 (27.4)
Alcohol consumption						
Harmful drinking	4,714 (0.7)	430 (1.3)	4,284 (0.7)	3,067 (3.8)	124 (5.2)	2,943 (3.7)
Higher risk drinking	686 (0.1)	35 (0.1)	651 (0.1)	899 (1.1)	18 (0.8)	881 (1.1)
Lower risk drinking	11,231 (1.7)	603 (1.9)	10,628 (1.7)	5,803 (7.1)	168 (7.0)	5,635 (7.1)
Previous higher risk/harmful drinking	90 (0)	<10** (0)	82 (0)	28 (0.0)	2 (0.1)	26 (0.0)
Zero alcohol	1,247 (0.2)	70 (0.2)	1,177 (0.2)	5,695 (7.0)	282 (11.8)	5,413 (6.8)
Missing	642,449 (97.3)	30,951 (96.4)	611,498 (97.3)	66,193 (81.0)	1,795 (75.1)	64,398 (81.2)

^*^FI scores of 0–0.12 = fit, >0.12–0.24 = mild frailty, >0.24–0.36 = moderate frailty, >0.36 = severe frailty. Not considered as a candidate predictor during model development.

^**^Exact values for small cell counts (<10) not reported due to SAIL Databank restrictions.

LQ, lower quartile; UQ, upper quartile; WIMD, Welsh Index of Multiple Deprivation; IMD, Indices of Multiple Deprivation

### Model development and internal validation

The eFalls prediction model is given in full in Supplementary Table S3.2. LASSO regression retained 75 predictors in the final model. Instability plots (Supplementary Figures S3.3/4) showed low variability in individual-level predictions and calibration curves, implying a stable model in the development population. Apparent performance, average optimism and optimism-adjusted estimates in the development dataset (not accounting for clustering by practice) are reported in Supplementary Table S3.3.

**Figure 1:**
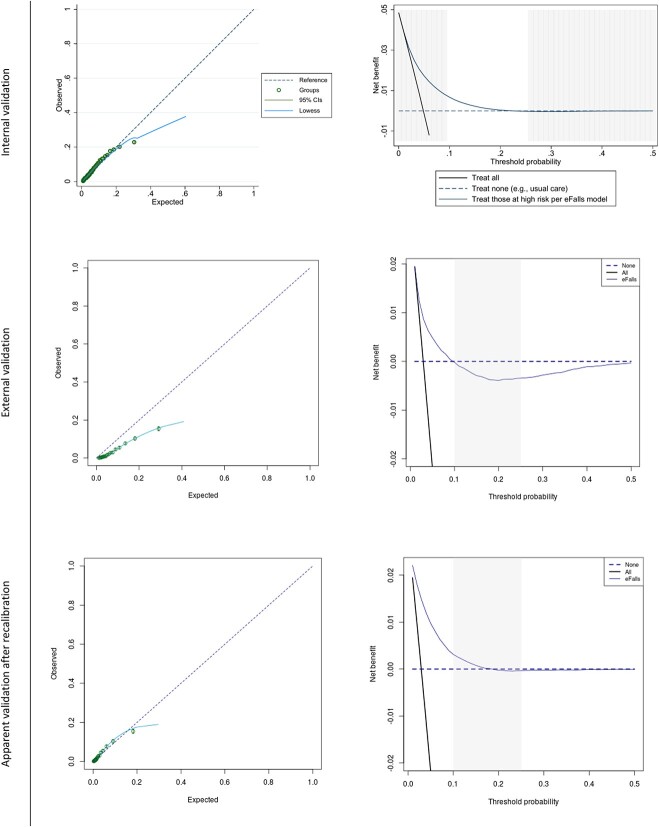
Calibration plots and decision curves of the eFalls model on internal validation (in the model development data) and external validation, before and after recalibration. The pre-defined region of clinical interest (threshold probabilities between 10% and 25%) is highlighted on the decision curves.

Apparent calibration in the model development data was excellent in the range of predicted risks up to 20%, with the calibration curve laying close to the diagonal line of ideal calibration (where predicted risks exactly match observed outcomes, see Figure 1). The calibration slope of 0.99 (95% confidence interval, CI: 0.75 to 1.22) and CITL of −0.13 (95% CI: −0.66 to 0.40) on internal–external cross-validation suggest good calibration, with some over-prediction of risks on average (see Table 2). Over-prediction was evident in the calibration plot for the 5% of the population with the highest predicted fall/fracture risk, with the summary point for this group lying below the diagonal on the calibration plot (in the region where predicted risks exceed observed outcomes). Calibration curves across general practices were variable, with over-prediction in some practices and under-prediction in others, as shown in Supplementary Figure S3.5.

**Table 2:** Predictive performance summary for the eFalls prediction model on internal validation, internal–external cross-validation and external validation, and the recalibrated model on apparent validation

	Performance in model development data		Performance in external validation data		Recalibrated model performance in external validation data	
	Pooled across GP practices (apparent)	Internal–external cross-validation	Overall performance	Pooled across GP practices	Overall performance (apparent)	Pooled across GP practices (apparent)
Calibration slope						
Summary estimate	0.99	0.99	1.248	1.203	1.000	0.964
95% confidence interval	0.97 to 1.01	0.75 to 1.22	1.244 to 1.265	1.133 to 1.273	1.000 to 1.013	0.908 to 1.020
95% prediction interval	0.80 to 1.18	0.30 to 1.67	–	0.858 to 1.548	–	0.687 to 1.240
${\tau}^2$ (95% CI)	0.009 (0.005 to 0.014)	0.052 (0.018 to 0.247)	–	0.029 (0.006 to 0.060)	–	0.018 (0.004 to 0.039)
Calibration-in-the-large						
Summary estimate	0.154	−0.13	−0.931	−0.874	0.000	0.064
95% confidence interval	0.095 to 0.212	−0.66 to 0.40	−0.938 to −0.920	−0.964 to −0.783	−0.009 to 0.010	−0.027 to 0.154
95% prediction interval	−0.96 to 1.27	−1.65 to 1.39	–	−1.375 to −0.372	–	−0.442 to 0.569
${\tau}^2$ (95% CI)	0.319 (0.275 to 0.373)	0.256 (0.091 to 1.219)	–	0.061 (0.035 to 0.151)	–	0.062 (0.035 to 0.149)
O/E ratio						
Summary estimate	1.19	0.88	0.432	0.431	1.000	1.013
95% confidence interval	1.12 to 1.26	0.53 to 1.46	0.430 to 0.437	0.388 to 0.479	0.992 to 1.009	0.916 to 1.122
95% prediction interval	0.14 to 2.24	0.21 to 3.70	–	0.194 to 0.958	–	0.475 to 2.163
${\tau}^2$ (95% CI)	0.282 (0.230 to 0.348)	0.228 (0.082 to 1.085)	–	0.157 (0.117 to 0.263)	–	0.142 (0.106 to 0.239)
C-statistic						
Summary estimate	0.72	0.72	0.825	0.816	0.825	0.816
95% confidence interval	0.72 to 0.72	0.68 to 0.76	0.824 to 0.828	0.801 to 0.830	0.825 to 0.828	0.801 to 1.000
95% prediction interval	0.68 to 0.76	0.59 to 0.85	–	0.715 to 0.886	–	0.715 to 0.886
${\tau}^2$ (95% CI)	0.010 (0.006 to 0.016)	0.002 (0.001 to 0.008)	–	0.078 (0.039 to 0.138)	–	0.078 (0.039 to 0.138)

**Figure 2:**
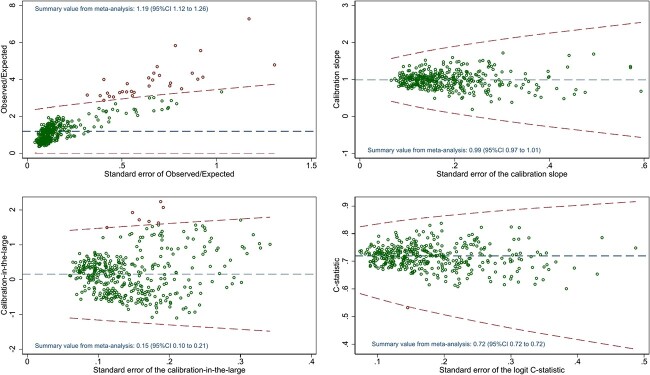
Variability in performance of the eFalls prediction model on internal validation, across GP practices in the model development data. Plots show calibration slope, calibration-in-the-large, observed/expected ratio and c-statistic plotted against their standard error within each practice. Bounds show 95% prediction intervals for the performance measure across possible standard errors.

C-statistics of 0.72 (95% CI: 0.72 to 0.72) when pooled across GP practices, and 0.72 (95% CI: 0.68 to 0.76) on internal–external cross-validation, show promising discrimination performance. As with calibration, discrimination performance varied across GP practice (Figure 2).

Decision curve analysis suggested that the model had clinical utility over treat-all and treat-none strategies in the model development data, with net benefit ranging from 0.008 (suggesting eight additional correctly identified falls/fracture events, over and above those falsely identified as being at high risk, per 1,000 older adults assessed using the model) down to 0 in the pre-specified risk range from 10% to 25% (Figure 1). While benefit (in terms of true positives) was seen in this range on average, the model did not exceed the next best strategy in all practices (Supplementary Figures S3.6/7).

Internal–external cross-validation showed consistent calibration and discrimination performance for models developed across WIMD subgroups (Supplementary Figures S3.8/9/10), except for in those missing WIMD details, where model performance was notably poor.

### External validation

The eFalls model was applied, as shown in Supplementary Box S3.1, to all individuals in Connected Bradford. Details of the prediction distributions in the external validation data are given in Supplementary Figure S.3.11.

**Figure 3:**
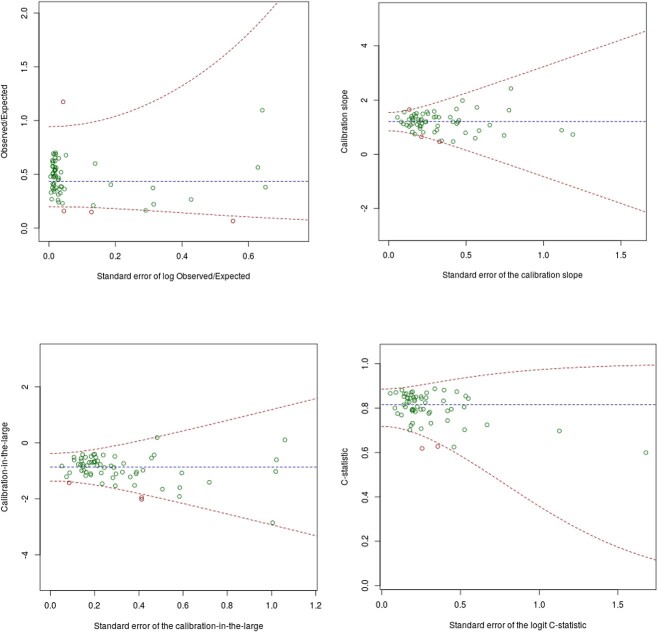
Variability in performance of the eFalls prediction model on external validation across GP practices—prior to recalibration. Plots show calibration slope, calibration-in-the-large, observed/expected ratio and c-statistic plotted against their standard error within each practice. Bounds show 95% prediction intervals for the performance measure across possible standard errors.

Discrimination performance on external validation was excellent, with a c-statistic of 0.816 (95% CI: 0.801 to 0.830) when pooled across practices (Table 2). The c-statistic was consistently above 0.6 in all practices, as shown in Figure 3, and was promising in most subgroups (Supplementary Table S3.4). When assessed across frailty groups, discrimination was notably lower in the severely frail subgroup, with a c-statistic of 0.643 (95% CI: 0.638 to 0.646), most likely due to the narrower case-mix distribution.

Calibration curves (Figure 1) show that the eFalls model over-predicted fall/fracture risk in the Connect Bradford population, with predicted risks exceeding observed risks across the full range of possible predicted values. This was confirmed by a pooled CITL estimate of −0.874 (95% CI: −0.964 to −0.783), which also suggested that predicted risks were too high on average. The extent of over-prediction was highly variable across GP practices (see Figures 3 and S3.12), but was seen consistently across subgroups by IMD, frailty, sex and BMI (Supplementary Figures S3.15–S3.18).

Thus, the eFalls model was recalibrated to better reflect the Connected Bradford population. Recalibration did not affect the discrimination performance or variability in calibration across practices (Supplementary Figures S3.12/S3.14), but corrected average over-prediction on apparent validation, giving a pooled calibration slope of 0.964 (95% CI: 0.908 to 1.020) and pooled CITL of 0.064 (95% CI: −0.027 to 0.154). The equation for the recalibrated model is provided in Supplementary Box S3.2.

Decision curves show that the eFalls model had clinical utility at lower threshold probabilities on external validation, but was no better than the treat-none alternative in the pre-specified range of 10–25%. Net benefit over clinically important thresholds did improve on average with recalibration, being superior to other strategies for threshold probabilities up to 18% (Figure 1). Supplementary Tables S3.4 and S3.5 give a breakdown of sensitivity and specificity at various threshold across this clinically important range, for the eFalls and the recalibrated models, respectively. As with calibration performance, net benefit was highly variable across GP practices, which did not stabilise with recalibration (Supplementary Figure S3.13).

Full details of model performance across clinically relevant subgroups are shown in Appendix S3. Model performance was fairly consistent across sexes and deprivation subgroups, while over-prediction of falls risk was most evident in those who were underweight and those who had severe frailty. The least net benefit was seen from using the model in those who were fit or had only mild frailty, where the proportion of fall/fracture events was lowest.

## Discussion

### Summary of main findings

This study developed and externally validated the eFalls prediction model, using routinely collected EHR data from around 750,000 older adults across two independent, retrospective cohorts. The eFalls model predicts the 1-year risk of a fall/fracture, with excellent discrimination on external validation (c-statistic 0.82, 95% CI: 0.80 to 0.83).

Calibration was variable across individual general practices in both the development and validation data, with a tendency for the model to over-predict fall/fracture risk in the Connected Bradford (external) population (O/E 0.43, 95% CI: 0.39 to 0.48). Over-prediction was present, though less extreme, on internal–external cross-validation across WIMD subgroups (O/E 0.88, 95% CI: 0.53 to 1.46), primarily in those with missing WIMD details.

Decision curve analysis in the development data suggested net benefit above treat-all and treat-none approaches when using the eFalls model with risk thresholds up 28%. This implies clinical utility when applying the eFalls model with thresholds between 10% and 25% for guiding decision making, which were specified a priori as being of clinical interest. On external validation, net benefit over other approaches was seen for lower threshold probabilities (below 10%) but was no better than treat-none for thresholds between 10% and 25%.

While the eFalls model was well calibrated on internal and internal–external cross-validation (in Welsh national-level data in SAIL), on external validation in Connected Bradford, although there was evidence for excellent discrimination, fall/fracture risk was slightly overestimated on average. This overestimation of risk was likely due to the higher incidence of falls in the development (4.9%) compared to the external validation cohorts (2.9%). Simple recalibration methods were employed, tailoring the eFalls model to the external validation data. This resulted in improvements to calibration performance in Connected Bradford (apparent calibration slope 0.964, 95% CI: 0.908 to 1.020; CITL 0.064, 95% CI: −0.027 to 0.154), while discrimination performance was unchanged (remaining excellent).

Such miscalibration is often the case when applying a prediction model in a new setting, due to differences between the two populations. In the UK, where populations across regions can vary greatly, there is a national programme to establish Secure Data Environments (SDEs), which will include regional NHS data. Given the current movement towards use of these regional SDEs following the Goldacre review [37], it is feasible (for the first time) for prediction models to be recalibrated on a regional basis. Thus, examining whether model recalibration notably improved calibration performance was considered important and relevant to contemporary practice. The performance of this recalibrated model was assessed only as apparent validation, thus its use is not currently recommended in practice without further validation.

Implementation of eFalls into primary care EHR systems could transform the way in which falls prevention services are organised and delivered, through a standardised method of efficient risk identification using existing primary care records without the need for resource intensive clinical assessments. For implementation, we would recommend use of the externally validated eFalls model, although our findings suggest that targeted recalibration to local or regional populations could also be beneficial. For example, a regional Integrated Care System in England may wish to examine recalibration options, where their routine health data infrastructure supports this, although those implementing such processes would need sufficient statistical knowledge around model recalibration and validation methods. The planned Secure Data Environments in England and related structures in the devolved nations could support this approach.

### Strengths and limitations of this work

Model development and external validation in two independent UK data resources from Wales and England, based on different systems and coding ontologies, is a notable strength of this study. This approach adds much-needed methodological rigour to the falls prediction field, which has hitherto been limited by the absence of external validation of models.

The population of interest in the study were frail older adults, thus there was a possibility of death precluding a fall/fracture during follow-up. In our modelling, we used logistic regression to estimate the 12-month event risk. Individuals that died during the 12-month period were retained in the risk set for the whole 12 months, so that risk estimates from our model correspond to a real-world situation where some individuals may die before experiencing the outcome of interest (and thus death precludes them from ever having the event). Hence, for some individuals predicted to have a low falls risk, this may in fact be due to a higher competing risk of death. An alternative approach could be a survival model accounting for the competing event of death, for example by using a Fine and Grey model [38]. Nevertheless, given the short time frame for prediction (just 12 months, with everyone followed for 12 months or until they died before 12 months) and low mortality rate during follow-up (3.8% in the model development data, 4.3% in the external validation data), we expect the impact of the modelling of the competing event of death to be low.

Regarding medications, many falls-risk-increasing medications have been identified previously, including anticholinergic medications, cardiovascular medications and gastrointestinal medications [39–41]. These were not included as individual candidate predictors in our model development, as this would have vastly increased the model complexity and required sample size. We instead chose to construct and include a single polypharmacy count variable, to be modelled using fractional polynomials to allow for non-linear associations with the outcome. This approach simplifies application of the model in new individuals, facilitating implementation in practice, while also capturing the prognostic effect of polypharmacy, which itself has also been reported as increasing falls risk [40].

Our extensive examination of the prediction model on internal validation, internal–external cross-validation and external validation included assessing important performance measures such as calibration, which have historically been overlooked in falls prediction work [13]. Use of decision curve analysis, with pre-defined thresholds of clinical interest, adds further methodological robustness and provides evidence of clinical utility for using eFalls in clinical practice to identify people who are at increased risk of experiencing a fall/fracture within the next year.

Due to differences in systems and coding ontologies across the model development and validation datasets, the eFalls model showed some miscalibration on external validation. Recalibration to the Connect Bradford population resulted in considerable improvements in calibration performance, while maintaining the high discrimination of the eFalls model.

We have not externally validated the eFalls prediction model using international data, which would be an important next step prior to wider implementation. It is also worth noting that the recalibrated model has not yet been tested externally, and the results presented here show the apparent calibration of the model (as applied in the data used to derive the recalibration). Differences in model calibration can stem from differences across populations; as such, the recalibrated model (tailored to the Connected Bradford data) would likely show poor calibration if applied to the population comprising the SAIL Databank.

### Comparison with previous literature

Numerous falls risk prediction models are now available in the literature, developed across a wide range of populations, though these have often not been externally validated and are at a high risk of bias [13]. Indeed, a 2021 systematic review of falls prediction models for community-dwelling older adults found that most studies included only very restricted populations due to their exclusion of individuals with falls-risk-increasing conditions. In comparison, the eFalls model included such individuals and considered these conditions as candidate predictors.

Only one external validation study had considered calibration performance at the time of the 2021 review, with calibration plots suggesting over-prediction of risk in new individuals and variation in calibration performance across populations [42], as was seen to be the case with the eFalls model. Similarly, a 2022 UK-based model developed and externally validated in EHR showed over-prediction of 1-year falls risk on external validation, with inconsistent calibration performance across GP practices [43]. Thus, the calibration of the eFalls model on external validation is consistent with other models in the area.

Discrimination performance of the eFalls model on internal validation was in line with that seen in other similar models (c-statistics ranging from 0.49 to 0.87 over 37 models) [13]. On external validation, discrimination performance was akin to that of a 2022 model, developed and validated only in those with an indication for antihypertensive treatment (pooled c-statistic across general practices: 0.866, 95% CI 0.862 to 0.869). When compared to models for more diverse community-dwelling populations, discrimination of the eFalls model was far superior (previously reported c-statistics from 0.62 to 0.69, 3 models).

### Implications for policy and practice

The use of routinely collected primary care EHR data for falls prediction is novel, with a historical absence of EHR-based falls prediction models developed and externally validated using rigorous prediction modelling methodology.

Using routinely collected data to identify individuals at high risk of falls, as opposed to using instruments that require additional clinical resources, is aligned with current requirements to support routine falls prevention service pathways. Using such an approach could also help support a more proactive approach to falls prevention, moving away from referral pathways predicated on an individual having experienced an initial fall event. The use of eFalls to identify people at high risk of experiencing fall/fracture events could further complement the development of new falls prevention services based around the World Falls Guideline algorithm.

Our next steps include seeking to implement the eFalls prediction model into UK primary care EHR systems, in partnership with system suppliers, and to discuss how local recalibration approaches might be implemented in practice. We aim to work in partnership with UK policymakers to explore how eFalls could be used to inform health policy in this important area and will seek to incorporate into future falls prevention guidelines. We will also seek to make eFalls available to Integrated Care Systems across England and the equivalent structures across the devolved nations. We will make the eFalls model available for future research into falls prevention and plan further external validation work in partnership with international researchers.

## Conclusion

The eFalls prediction model has good predictive performance and could support proactive stratification as part of falls prevention services, if appropriately embedded into primary care EHR systems, including local recalibration where possible. This could help transform how falls prevention services are organised and delivered in the UK and, in the future, internationally.

## Supplementary Material

aa-23-2211-File002_afae057

## Data Availability

The eFalls model equation as published in this manuscript is available for research use. Code lists used to define variables are available on reasonable request from the corresponding author. We will make eFalls available to suppliers of UK electronic health record systems, risk stratification software, and for use in NHS policy and commissioning under the terms of an agreed licence agreement. Any unauthorised use or distribution for commercial purposes is forbidden.
