# Supplementary material for: Development and external validation of the eFalls tool: a multivariable prediction model for the risk of ED attendance or hospitalisation with a fall or fracture in older adults
Source: Age Ageing. 2024 Mar 22;53(3):afae057. doi: 10.1093/ageing/afae057 (PMC10960070; doi:10.1093/ageing/afae057)
Supplement: aa-23-2211-File002_afae057 [file aa-23-2211-file002_afae057.docx]

Development and external validation of the eFalls tool: a multivariable prediction model for the risk of ED attendance or hospitalisation with a fall or fracture in older adults - Supplementary Materials

Data availability and intellectual property

The eFalls model equation as published in this manuscript is available for research use. Code lists used to define variables are available from the corresponding author. We will make eFalls available to suppliers of UK electronic health record systems, risk stratification software, and for use in NHS policy and commissioning under the terms of an agreed license agreement. Any unauthorised use or distribution for commercial purposes is forbidden.

# Appendix I – TRIPOD-Cluster checklist

#### Table S1.1: TRIPOD-Cluster checklist of items to include when reporting a study developing or validating a multivariable prediction model using clustered data (1)

| # | Description | Page # |
| --- | --- | --- |
| Title and abstract | |  |
| 1 | Identify the study as developing and/or validating a multivariable prediction model, the target population, and the outcome to be predicted | Page 1 |
| 2 | Provide a summary of research objectives, setting, participants, data source, sample size, predictors, outcome, statistical analysis, results, and conclusions.* | Page 3 |
|  | |  |
| Introduction | |  |
| 3a | Explain the medical context (including whether diagnostic or prognostic) and rationale for developing or validating the prediction model, including references to existing models, and the advantages of the study design.* | Page 4 |
| 3b | Specify the objectives, including whether the study describes the development or validation of the model.* | Page 5 |
|  |  |  |
| Methods |  |  |
| 4a | Describe eligibility criteria for participants and datasets.* | Page 6 (Population),  Figure 1 |
| 4b | Describe the origin of the data, and how the data were identified, requested, and collected. | Page 6 (Methods, Population) |
| 5 | Explain how the sample size was arrived at.* | Page 7 (Sample size)  Table S2.1  Table S2.2 |
| 6a | Define the outcome that is predicted by the model, including how and when assessed.* | Page 6 (Outcome) |
| 6b | Define all predictors used in developing or validating the model, including how and when measured.* | Supp page 2 (Data availability and intellectual property) |
| 7a | Describe how the data were prepared for analysis, including any cleaning, harmonisation, linkage, and quality checks. |  |
| 7b | Describe the method for assessing risk of bias and applicability in the individual clusters (e.g., using PROBAST). | NA – clustering at GP level |
| 7c | For validation, identify any differences in definition and measurement from the development data (e.g., setting, eligibility criteria, outcome, predictors).* | Page 6 (Outcomes, Predictors) |
| 7d | Describe how missing data were handled.* | Page 7 (Missing data) |
| 8a | Describe how predictors were handled in the analyses. | Page 8 (Model development) |
| 8b | Specify the type of model, all model-building procedures (e.g., any predictor selection and penalisation), and method for validation.* | Page 8 (Model development) |
| 8c | Describe how any heterogeneity across clusters (e.g., studies or settings) in model parameter values was handled. | Page 8 (Model development) |
| 8d | For validation, describe how the predictions were calculated. | Page 9 (External validation),  Box S3.1 |
| 8e | Specify all measures used to assess model performance (e.g., calibration, discrimination, and decision curve analysis) and, if relevant, to compare multiple models. | Page 8 (Internal validation) |
| 8f | Describe how any heterogeneity across clusters (e.g., studies or settings) in model performance was handled and quantified. | Page 8 (Internal validation) |
| 8g | Describe any model updating (e.g., recalibration) arising from the validation, either overall or for particular populations or settings.* | Page 9 (External validation) |
| 9 | Describe any planned subgroup or sensitivity analysis, (e.g., assessing performance according to sources of bias, participant characteristics, setting). | Page 9 (External validation), |
|  |  |  |
| Results |  |  |
| 10a | Describe the number of clusters and participants from data identified through to data analysed. A flow chart may be helpful.* | Page 10 (Summary of development and validation datasets) |
| 10b | Report the characteristics overall and where applicable for each data source or setting, including the key dates, predictors, treatments received, sample size, number of outcome events, follow-up time, and amount of missing data.* | Table 1,  Table S3.1 |
| 10c | For validation, show a comparison with the development data of the distribution of important variables (demographics, predictors, and outcome). | Table 1,  Table S3.1 |
| 11 | Report the results of the risk of bias assessment in the individual clusters. | NA – clustering at GP level |
| 12a | Report the results of any across-cluster heterogeneity assessments that led to subsequent actions during the model’s development (e.g., inclusion or exclusion of particular predictors or clusters). | Figure 2  Table 2 (tau, PI)  Figure S3.5/6/7 |
| 12b | Present the final prediction model (i.e., all regression coefficients, and model intercept or baseline estimate of the outcome at a given time point) and explain how to use it for predictions in new individuals.* | Table S3.2,  Box S3.1 |
| 13a | Report performance measures (with uncertainty intervals) for the prediction model, overall and for each cluster. | Overall: Table 2/S3.2  Cluster specific: scatterplots only due to large number of clusters. Figure 2/3. |
| 13b | Report results of any heterogeneity across clusters in model performance. | Table 2,  Figure 3 |
| 14 | Report the results from any model updating (including the updated model equation and subsequent performance), overall and for each cluster.* | Overall: Table 2, Box S3.2  Cluster specific: scatterplots only due to large number of clusters. Figure S3.12/13/14. |
| 15 | Report results from any subgroup or sensitivity analysis. | Table S3.4,  Figure S3.15-18 |
|  | |  |
| Discussion | |  |
| 16a | Give an overall interpretation of the main results, including heterogeneity across clusters in model performance, in the context of the objectives and previous studies.* | Page 19 (Summary of main findings) |
| 16b | For validation, discuss the results with reference to the model performance in the development data, and in any previous validations. | Page 19 (Summary of main findings) |
| 16c | Discuss the strengths of the study and any limitations (e.g., missing or incomplete data, nonrepresentativeness, data harmonisation problems).* | Page 19/20 (Strengths and limitations of this work) |
| 17 | Discuss the potential use of the model and implications for future research, with specific view to generalisability and applicability of the model across different settings or (sub)populations.* | Page 20/21 (Implications for policy and practice, Conclusion) |
|  | |  |
| Other information | |  |
| 18 | Provide information about the availability of supplementary resources (e.g., study protocol, analysis code, datasets).* | Not yet available. To be included at publication. |
| 19 | Give the source of funding and the role of the funders for the present study. | Page 1 (Title page) |

This checklist is taken from Debray TPA, Collins GS, Riley RD et al. Transparent reporting of multivariable prediction models developed or validated using clustered data: TRIPOD-Cluster checklist. BMJ 2022;378:e071018; doi:10.1136/bmj2022-071018.

PROBAST=prediction model risk-of-bias assessment tool.

*Item text is an adaptation of one or more existing items from the original TRIPOD (transparent reporting of a multivariable prediction model for individual prognosis or diagnosis) checklist.

# Appendix 2 – extended methods section

## Detailed sample size calculations

#### Table S2.1: Sample size requirements for model development, based on Riley et al 2019 criteria (2)

| Outcome | Prevalence | Parameters | Assumed Nagelkerke $R^{2}$ | Source for $R^{2}$ | Required sample size,  n (events) |
| --- | --- | --- | --- | --- | --- |
| Fall/fracture | 4.8% | 90 | 0.15 | Riley et al (2) | 13867 (666) |
|  | 4.8% | 90 | 0.05 | $R^{2}$, original eFI (3) | 50174 (2409) |
|  | 4.8% | 90 | 0.049 | $C$, original eFI (3) | 50927 (2445) |

#### Table S2.2: Sample size requirements for model validation, based on Riley et al 2021 criteria (4)

|  | Assumptions | Required sample size, n (events) |
| --- | --- | --- |
| O/E | Outcome prevalence: 0.048 | 7625 (366) |
| Calibration slope | Outcome prevalence: 0.048  Linear predictor distribution*:  Mean: -3.30  Variance: 0.690  Skew: 0.5  Kurtosis: 3 | 10882 (523) |
| C-statistic | Outcome prevalence: 0.048  C-statistic: 0.743 | 2027 (98) |
| Standardised net benefit (10%) | Outcome prevalence: 0.048  Sensitivity: 0.34  Specificity: 0.91 | 2289 (110) |
| Standardised net benefit (25%) | Outcome prevalence: 0.048  Sensitivity: 0.04  Specificity: 0.99 | 519 (25) |
| Overall | (as above) | 10882 (523) |

* Note in the model development data linear predictor, skew and kurtosis were recorded as 0.499 and 2.943 respectively. The closest available pairs for skewed normal distribution generation in the Stata package *sknor* were assessed, and the most conservative option (giving the largest required minimum sample size) was chosen.

## Detailed outcome definition

#### Table S2.3: ICD10 codes used to define ED attendance or hospital admission for a fall or fracture

| Code | Description |
| --- | --- |
| W00 | Fall on same level involving ice and snow |
| W01 | Fall on same level from slipping, tripping and stumbling |
| W02 | Fall involving ice-skates, skis, roller-skates or skateboards |
| W03 | Other fall on same level due to collision with, or pushing by, another person |
| W04 | Fall while being carried or supported by other persons |
| W05 | Fall involving wheelchair |
| W06 | Fall involving bed |
| W07 | Fall involving chair |
| W08 | Fall involving other furniture |
| W09 | Fall involving playground equipment |
| W10 | Fall on and from stairs and steps |
| W11 | Fall on and from ladder |
| W12 | Fall on and from scaffolding |
| W13 | Fall from, out of or through building or structure |
| W14 | Fall from tree |
| W15 | Fall from cliff |
| W16 | Diving or jumping into water causing injury other than drowning or submersion |
| W17 | Other fall from one level to another |
| W18 | Other fall on same level |
| W19 | Unspecified fall |
| M80 | Osteoporosis with pathological fracture |
| S22 | Fracture of rib(s), sternum and thoracic spine |
| S32 | Fracture of lumbar spine and pelvis |
| S42 | Fracture of shoulder and upper arm |
| S52 | Fracture of forearm |
| S72 | Fracture of femur |
| S82 | Fracture of lower leg, including ankle |
| T08 | Fracture of spine, level unspecified |
| T10 | Fracture of upper limb, level unspecified |
| T12 | Fracture of lower limb, level unspecified |
| T14.2 | Fracture of unspecified body region |

## Recalibration procedure

Applying a prediction model in a new dataset that involves a different population to that of model development often results in different predictive performance due to changes in demographics, case-mix, incidence rates, etc. One method to address these differences when applying the model in a new population is recalibration. Recalibration involves adjusting the original model to better fit the new data, tailoring it to the new case-mix.

Recalibration methods involve varying levels of complexity, from simple intercept adjustments for changes in baseline risk, to re-estimation of coefficients and addition of new predictor parameters. By design, this recalibrated model is optimally calibrated to this new data and would be poorly calibrated if applied back in the original model development data. A recalibrated model may be better suited to a new population similar to the recalibration dataset, but requires additional external validation to assess its suitability, just as an entirely new model would.

Given the current direction of travel towards regional Secure Data Environments (SDEs) following the 2022 Goldacre review (5), it is feasible for models to be recalibrated on a regional basis in the future. This would allow for the application of models tailored to specific populations, which are then well calibrated to their own setting, rather than attempts to apply a “one size fits all” model across diverse regions.

To case-test this concept, we performed a simple method of recalibration which adjusted only the intercept and slope of the model, retaining the relative weighting between coefficients, thus maintaining the overall structure of the overarching eFalls model. This method of recalibration maintained the ordering patients, with respect to predicted risk, and so did not alter discrimination performance. Improvements to model calibration, however, leads to predictions that better reflect the magnitude of fall/fracture risk in an individual from that population.

When applying the original model in the new population, a value for the linear predictor, $LP_{eFalls_{i}}$, is obtained for each individual, $i$, based on a combination of their characteristics and the model coefficient and intercept values from the eFalls model. The process of calculating these values is shown in Box S3.1 in Appendix 3.2, below.

To recalibrate the model, a logistic regression model is fit to predict the fall/fracture outcome ($Y_{i}$, say) in the new data, with the only parameters being the intercept term and a coefficient for the value of ${LP}_{eFalls_{i}}$, such that:

$$ln(\frac{P_{recal}}{1-P_{recal}})=\alpha_{recal}+\beta_{recal}(LP_{eFalls})$$

where $P_{recal}$ gives the predicted fall/fracture probability from the recalibrated model, $\alpha_{recal}$ gives the change to the intercept due to differing baseline risk, and $\beta_{recal}$ gives the change to the slope of the model to better reflect the dispersion of risk within the new population. The application of this recalibrated model is shown in Box S3.2 in Appendix 3.4. This model was fit using the glm command in R (version 4.2.3).

# Appendix 3 – extended results

## Population characteristics

#### Table S3.1 Further descriptive statistics (frailty deficits) for model development and external validation cohorts, stratified by outcome status at 12 months

|  | Model development data | | | External validation data | | |
| --- | --- | --- | --- | --- | --- | --- |
|  | Total | Fall/fracture | No fall/fracture | Total | Fall/fracture | No fall/fracture |
| *n* | *660,417* | *32,097 (4.9)* | *628,320 (95.1)* | *81,685* | *2,389 (2.9)* | *79,296 (97.1)* |
|  |  |  |  |  |  |  |
| Abdominal pain | 101210 (15.3) | 6286 (19.6) | 94924 (15.1) | 15393 (18.8) | 468 (19.6) | 14925 (18.8) |
| Activity limitation | 5646 (0.9) | 495 (1.5) | 5151 (0.8) | 1689 (2.1) | 224 (9.4) | 1465 (1.8) |
| Anaemia and haematinic deficiency | 57016 (8.6) | 5365 (16.7) | 51651 (8.2) | 13356 (16.4) | 727 (30.4) | 12629 (15.9) |
| Anxiety* | 32014 (4.8) | 2457 (7.7) | 29557 (4.7) | 5838 (7.1) | 282 (11.8) | 5556 (7.0) |
| Asthma | 135836 (20.6) | 9272 (28.9) | 126564 (20.1) | 15812 (19.4) | 556 (23.3) | 15256 (19.2) |
| Atrial fibrillation | 59098 (8.9) | 5351 (16.7) | 53747 (8.6) | 11690 (14.3) | 662 (27.7) | 11028 (13.9) |
| Back pain | 181488 (27.5) | 12132 (37.8) | 169356 (27) | 24164 (29.6) | 789 (33.0) | 23375 (29.5) |
| Bone disease | 5574 (0.8) | 483 (1.5) | 5091 (0.8) | 7831 (9.6) | 329 (13.8) | 7502 (9.5) |
| Cancer | 134167 (20.3) | 8689 (27.1) | 125478 (20) | 21378 (26.2) | 776 (32.5) | 20602 (26.0) |
| Chronic kidney disease* | 156983 (23.8) | 11666 (36.3) | 145317 (23.1) | 35713 (43.7) | 1396 (58.4) | 34317 (43.3) |
| Cognitive impairment | 6644 (1) | 842 (2.6) | 5802 (0.9) | 26706 (32.7) | 917 (38.4) | 25789 (32.5) |
| COPD | 77849 (11.8) | 5820 (18.1) | 72029 (11.5) | 10730 (13.1) | 479 (20.1) | 10251 (12.9) |
| Dementia | 18870 (2.9) | 2601 (8.1) | 16269 (2.6) | 8597 (10.5) | 762 (31.9) | 7835 (9.9) |
| Depression | 43299 (6.6) | 3793 (11.8) | 39506 (6.3) | 8321 (10.2) | 456 (19.1) | 7865 (9.9) |
| Diabetes mellitus | 124173 (18.8) | 8311 (25.9) | 115862 (18.4) | 21157 (25.9) | 741 (31.0) | 20416 (25.7) |
| Dizziness | 88020 (13.3) | 6320 (19.7) | 81700 (13) | 11112 (13.6) | 444 (18.6) | 10668 (13.5) |
| Dressing and grooming problems | <10 (0) | <10 (0) | <10 (0) | 12277 (15.0) | 852 (35.7) | 11425 (14.4) |
| Dyspnoea* | 68751 (10.4) | 5657 (17.6) | 63094 (10) | 15099 (18.5) | 702 (29.4) | 14397 (18.2) |
| Environment problems* | 12249 (1.9) | 1264 (3.9) | 10985 (1.7) | 3054 (3.7) | 273 (11.4) | 2781 (3.5) |
| Faecal incontinence | 5589 (0.8) | 509 (1.6) | 5080 (0.8) | 3824 (4.7) | 315 (13.2) | 3509 (4.4) |
| Falls | 106839 (16.2) | 10513 (32.8) | 96326 (15.3) | 18182 (22.3) | 1855 (77.6) | 16327 (20.6) |
| Fatigue | 23641 (3.6) | 1725 (5.4) | 21916 (3.5) | 5348 (6.5) | 255 (10.7) | 5093 (6.4) |
| Foot problems | 51706 (7.8) | 4159 (13) | 47547 (7.6) | 14499 (17.7) | 644 (27.0) | 13855 (17.5) |
| Fracture | 157004 (23.8) | 11342 (35.3) | 145662 (23.2) | 19453 (23.8) | 1256 (52.6) | 18197 (22.9) |
| Fragility fracture | 79033 (12) | 7586 (23.6) | 71447 (11.4) | 15018 (18.4) | 1170 (49.0) | 13848 (17.5) |
| General mental health | 44450 (6.7) | 4300 (13.4) | 40150 (6.4) | 18079 (22.1) | 1397 (58.5) | 16682 (21.0) |
| Headache | 34383 (5.2) | 2255 (7) | 32128 (5.1) | 4134 (5.1) | 133 (5.6) | 4001 (5.0) |
| Hearing impairment | 122237 (18.5) | 8177 (25.5) | 114060 (18.2) | 18685 (22.9) | 760 (31.8) | 17925 (22.6) |
| Heart failure | 76939 (11.7) | 5957 (18.6) | 70982 (11.3) | 8915 (10.9) | 519 (21.7) | 8396 (10.6) |
| Heart valve disease* | 24071 (3.6) | 2069 (6.4) | 22002 (3.5) | 6296 (7.7) | 281 (11.8) | 6015 (7.6) |
| Housebound | 74800 (11.3) | 9035 (28.1) | 65765 (10.5) | 8931 (10.9) | 931 (34.8) | 8100 (10.2) |
| Hypertension | 347534 (52.6) | 20486 (63.8) | 327048 (52.1) | 54726 (67.0) | 1839 (77.0) | 52887 (66.7) |
| Hypotension or syncope | 37756 (5.7) | 3568 (11.1) | 34188 (5.4) | 14582 (17.9) | 975 (40.8) | 13607 (17.2) |
| Inflammatory arthritis | 96213 (14.6) | 6424 (20) | 89789 (14.3) | 17549 (21.5) | 625 (26.2) | 16924 (21.3) |
| Inflammatory bowel disease | 30168 (4.6) | 2074 (6.5) | 28094 (4.5) | 1315 (1.6) | 40 (1.7) | 1275 (1.6) |
| Ischaemic heart disease* | 97668 (14.8) | 7050 (22) | 90618 (14.4) | 22880 (28.0) | 990 (41.4) | 21890 (27.6) |
| Liver problems | 3400 (0.5) | 312 (1) | 3088 (0.5) | 1112 (1.4) | 35 (1.5) | 1077 (1.4) |
| Meal preparation problems | 298 (0) | 25 (0.1) | 273 (0) | 1106 (1.4) | 159 (6.7) | 947 (1.2) |
| Medication management | <10 (0) | <10 (0) | <10 (0) | 480 (0.6) | 54 (2.3) | 426 (0.5) |
| Memory concerns | 25552 (3.9) | 3307 (10.3) | 22245 (3.5) | 885 (1.1) | 28 (1.2) | 857 (1.1) |
| Mobility problems | 8894 (1.3) | 973 (3) | 7921 (1.3) | 15805 (19.3) | 1481 (62.0) | 14324 (18.1) |
| Mono or hemiparesis | 8102 (1.2) | 737 (2.3) | 7365 (1.2) | 1342 (1.6) | 82 (3.4) | 1260 (1.6) |
| Motor neurone disease | 251 (0) | 14 (0) | 237 (0) | 91 (0.1) | 6 (0.3) | 85 (0.1) |
| Musculoskeletal problems | 236264 (35.8) | 15129 (47.1) | 221135 (35.2) | 29156 (35.7) | 919 (38.5) | 28237 (35.6) |
| Osteoarthritis | 220727 (33.4) | 14994 (46.7) | 205733 (32.7) | 33211 (40.7) | 1173 (49.1) | 32038 (40.4) |
| Osteoporosis | 78236 (11.8) | 7591 (23.7) | 70645 (11.2) | 14915 (18.3) | 941 (39.4) | 13974 (17.6) |
| Palliative care | 5451 (0.8) | 484 (1.5) | 4967 (0.8) | 5584 (6.8) | 472 (19.8) | 5112 (6.4) |
| Parkinsonism and tremor | 20670 (3.1) | 2043 (6.4) | 18627 (3) | 2537 (3.1) | 177 (7.4) | 2360 (3.0) |
| Peptic ulcer disease | 4941 (0.7) | 365 (1.1) | 4576 (0.7) | 979 (1.2) | 38 (1.6) | 941 (1.2) |
| Peripheral neuropathy | 44734 (6.8) | 3217 (10) | 41517 (6.6) | 8797 (10.8) | 424 (17.7) | 8373 (10.6) |
| Peripheral vascular disease | 44496 (6.7) | 3578 (11.1) | 40918 (6.5) | 10952 (13.4) | 686 (28.7) | 10266 (12.9) |
| Problems managing finances* | 227 (0) | 16 (0) | 211 (0) | 4 (0.0) | 0 (0.0) | 4 (0.0) |
| Requirement for care | 23427 (3.5) | 2149 (6.7) | 21278 (3.4) | 6689 (8.2) | 519 (21.7) | 6170 (7.8) |
| Respiratory disease | 58989 (8.9) | 4260 (13.3) | 54729 (8.7) | 8783 (10.8) | 415 (17.4) | 8368 (10.6) |
| Seizures | 15238 (2.3) | 1314 (4.1) | 13924 (2.2) | 2171 (2.7) | 134 (5.6) | 2037 (2.6) |
| Self-harm | 1601 (0.2) | 157 (0.5) | 1444 (0.2) | 623 (0.8) | 48 (2.0) | 575 (0.7) |
| Severe mental illness | 46665 (7.1) | 4385 (13.7) | 42280 (6.7) | 15873 (19.4) | 767 (32.1) | 15106 (19.1) |
| Shopping problems* | 302 (0) | 28 (0.1) | 274 (0) | 473 (0.6) | 64 (2.7) | 409 (0.5) |
| Skin ulcer | 75506 (11.4) | 5735 (17.9) | 69771 (11.1) | 10231 (12.5) | 869 (36.4) | 9362 (11.8) |
| Sleep problems | 22554 (3.4) | 1815 (5.7) | 20739 (3.3) | 3765 (4.6) | 186 (7.8) | 3579 (4.5) |
| Social vulnerability | 20681 (3.1) | 1747 (5.4) | 18934 (3) | 10331 (12.6) | 815 (34.1) | 9516 (12.0) |
| Stress | 15374 (2.3) | 962 (3) | 14412 (2.3) | 3049 (3.7) | 163 (6.8) | 2886 (3.6) |
| Stroke | 48091 (7.3) | 4158 (13) | 43933 (7) | 10733 (13.1) | 633 (26.5) | 10100 (12.7) |
| Thyroid problems | 108866 (16.5) | 7483 (23.3) | 101383 (16.1) | 8765 (10.7) | 347 (14.5) | 8418 (10.6) |
| Toileting problems* | 241 (0) | 18 (0.1) | 223 (0) | 2401 (2.9) | 277 (11.6) | 2124 (2.7) |
| Transient ischaemic attack* | 29018 (4.4) | 2506 (7.8) | 26512 (4.2) | 4911 (6.0) | 281 (11.8) | 4630 (5.8) |
| Urinary incontinence | 41496 (6.3) | 3448 (10.7) | 38048 (6.1) | 13310 (16.3) | 858 (35.9) | 12452 (15.7) |
| Urinary system disease | 155282 (23.5) | 10391 (32.4) | 144891 (23.1) | 32769 (40.1) | 1656 (69.3) | 31113 (39.2) |
| Visual impairment | 145462 (22) | 11310 (35.2) | 134152 (21.4) | 34017 (41.6) | 1374 (57.5) | 32643 (41.2) |
| Washing and bathing | 377 (0.1) | 33 (0.1) | 344 (0.1) | 2796 (3.4) | 356 (14.9) | 2440 (3.1) |
| Weakness | 792 (0.1) | 69 (0.2) | 723 (0.1) | 1120 (1.4) | 89 (3.7) | 1031 (1.3) |
| Weight loss | 49466 (7.5) | 4141 (12.9) | 45325 (7.2) | 6114 (7.5) | 474 (19.8) | 5640 (7.1) |

*Considered as a candidate predictor, but omitted from the model during the LASSO selection process

**Exact values for small cell counts (<10) not reported due to SAIL Databank restrictions.

## Model development

#### Figure S3.1 Best fitting functional forms of continuous predictor variables (age and polypharmacy), after adjusting for other covariates in the complete model (no variable selection)

| 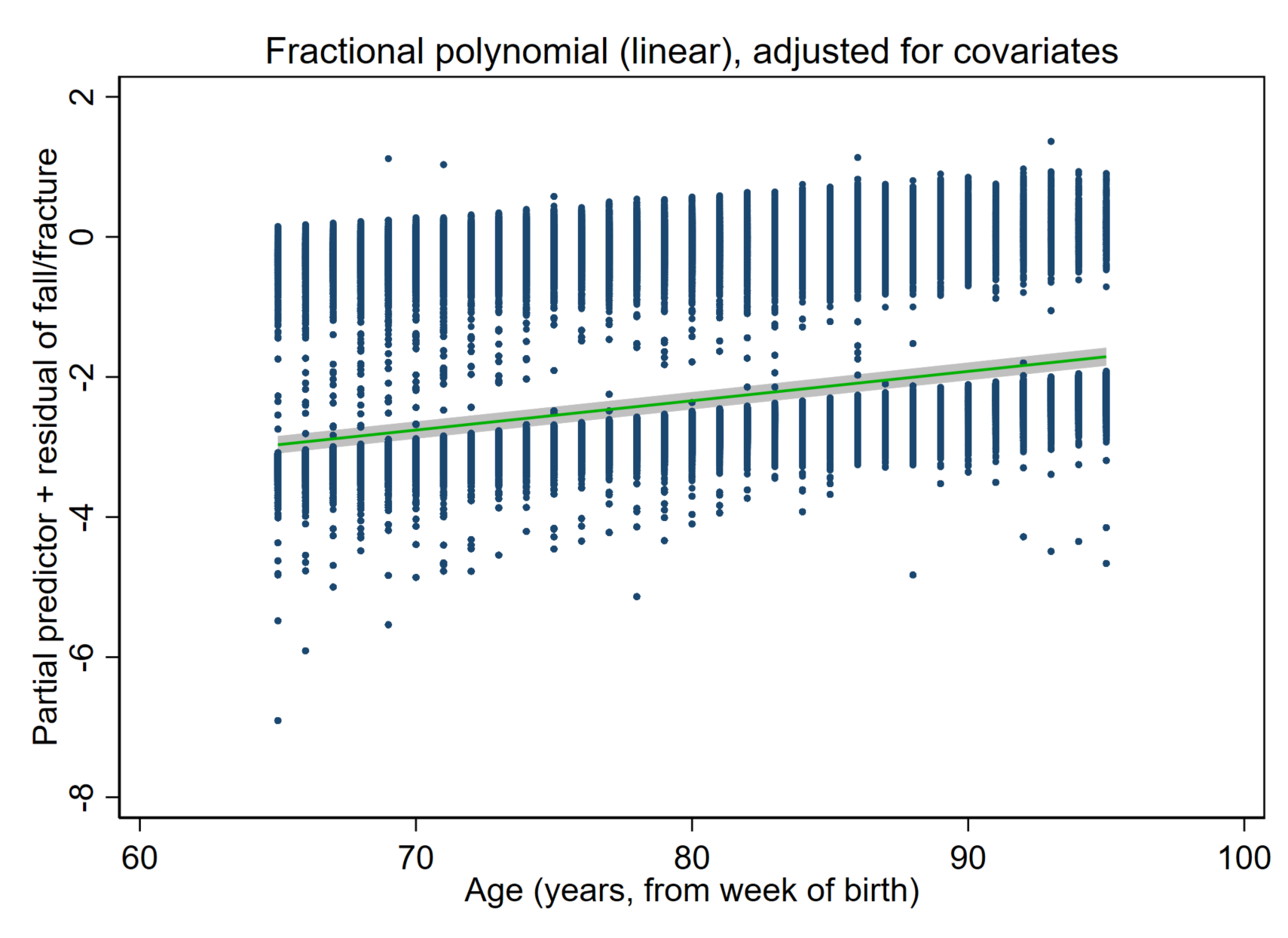 |
| --- |
| 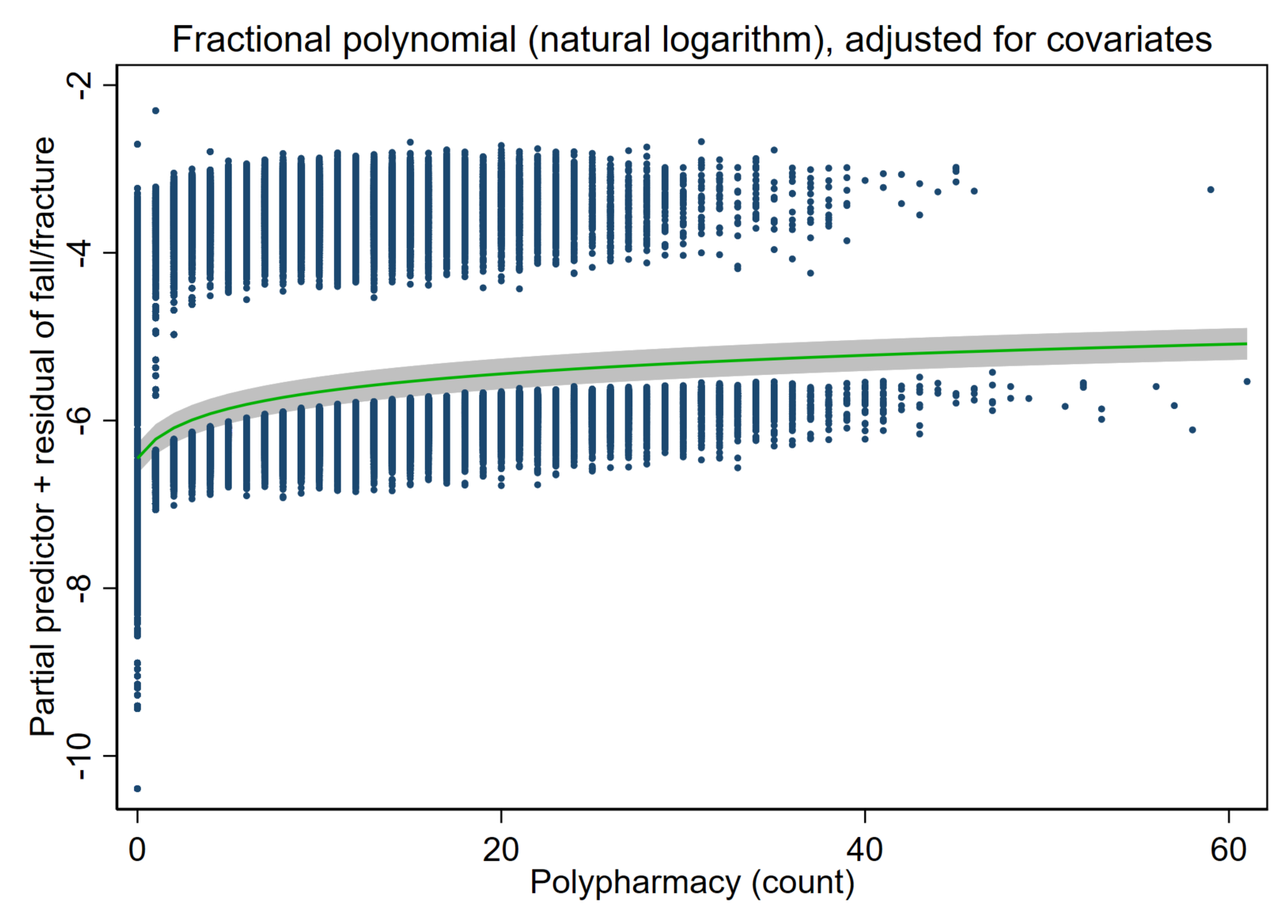 |

#### Table S3.2: Model coefficients and unpenalised model, giving predictor effects as odds ratios with 95% confidence intervals for all predictors included in the LASSO model (unpenalised logistic model refit with only LASSO selected predictor variables)

| Variable | Final penalised model,  Coefficient | Unpenalised model,  Odds Ratio (95% confidence interval) |
| --- | --- | --- |
|  |  |  |
| Age (years) | 0.0415506 | 1.043 (1.041 to 1.045) |
|  |  |  |
| Polypharmacy |  |  |
| ln((Polypharmacy+1)/10) | 0.3296295 | 1.392 (1.366 to 1.418) |
|  |  |  |
| Gender |  |  |
| Male | Reference |  |
| Female | -0.303708 | 0.732 (0.712 to 0.753) |
|  |  |  |
| BMI category |  |  |
| Underweight | 0.4896735 | 1.632 (1.537 to 1.732) |
| Normal | 0.2394177 | 1.269 (1.228 to 1.31) |
| Overweight | Reference |  |
| Obese | -0.0411134 | 0.952 (0.92 to 0.985) |
| Missing | -0.1451981 | 0.858 (0.825 to 0.892) |
|  |  |  |
| Smoking |  |  |
| Ex/never | Reference |  |
| Current | 0.0684529 | 1.078 (1.039 to 1.118) |
|  |  |  |
| Alcohol consumption |  |  |
| Harmful drinking | 0.4164064 | 1.536 (1.343 to 1.756) |
| Higher risk drinking | 0.1549725 | 1.255 (0.879 to 1.791) |
| Lower risk drinking | Reference |  |
| Previous higher risk/harmful drinking | 0.0849676 | 1.226 (0.577 to 2.603) |
| Zero alcohol | 0.0070124 | 1.055 (0.812 to 1.369) |
| Missing | -0.0679367 | 0.935 (0.858 to 1.018) |
|  |  |  |
| Abdominal pain | -0.0641861 | 0.93 (0.902 to 0.959) |
| Activity limitation | 0.0475092 | 1.064 (0.966 to 1.172) |
| Anaemia and haematinic deficiency | 0.1733029 | 1.193 (1.153 to 1.234) |
| Asthma | 0.0816046 | 1.086 (1.052 to 1.121) |
| Atrial fibrillation | 0.1519654 | 1.17 (1.131 to 1.21) |
| Back pain | 0.0498699 | 1.054 (1.027 to 1.081) |
| Bone disease | -0.0267845 | 0.956 (0.867 to 1.054) |
| Cancer | 0.0301854 | 1.035 (1.007 to 1.063) |
| Cognitive impairment | 0.1472251 | 1.168 (1.079 to 1.264) |
| COPD | 0.039956 | 1.042 (1.007 to 1.08) |
| Dementia | 0.1038111 | 1.116 (1.054 to 1.181) |
| Depression | 0.1633415 | 1.182 (1.136 to 1.229) |
| Diabetes mellitus | 0.0373911 | 1.042 (1.01 to 1.075) |
| Dizziness | 0.0198363 | 1.024 (0.993 to 1.055) |
| Dressing and grooming problems | 0.4532777 | 1.867 (0.374 to 9.326) |
| Faecal incontinence | -0.071791 | 0.914 (0.83 to 1.007) |
| Falls | 0.3009161 | 1.351 (1.314 to 1.389) |
| Fatigue | -0.0635057 | 0.928 (0.88 to 0.978) |
| Foot problems | 0.0282736 | 1.031 (0.993 to 1.069) |
| Fracture | 0.1957923 | 1.219 (1.187 to 1.253) |
| Fragility fracture | 0.2031303 | 1.226 (1.186 to 1.266) |
| General mental health | 0.0991068 | 1.107 (1.06 to 1.155) |
| Headache | -0.0149365 | 0.975 (0.931 to 1.022) |
| Hearing impairment | -0.0168728 | 0.976 (0.949 to 1.003) |
| Heart failure | -0.0190399 | 0.971 (0.94 to 1.004) |
| Housebound | 0.2549983 | 1.289 (1.25 to 1.329) |
| Hypertension | -0.0318888 | 0.959 (0.934 to 0.986) |
| Hypotension or syncope | 0.0778591 | 1.084 (1.046 to 1.122) |
| Inflammatory arthritis | 0.0586785 | 1.065 (1.034 to 1.098) |
| Inflammatory bowel disease | 0.0145135 | 1.021 (0.973 to 1.072) |
| Liver problems | 0.3803626 | 1.485 (1.314 to 1.677) |
| Meal preparation problems | -0.140024 | 0.834 (0.513 to 1.357) |
| Medication management | 0.8030273 | 3.153 (0.318 to 31.278) |
| Memory concerns | 0.2601186 | 1.296 (1.238 to 1.358) |
| Mobility problems | -0.1310067 | 0.865 (0.805 to 0.929) |
| Mono or hemiparesis | 0.1020457 | 1.119 (1.032 to 1.213) |
| Motor neurone disease | -0.1209976 | 0.805 (0.464 to 1.398) |
| Musculoskeletal problems | 0.0419361 | 1.046 (1.019 to 1.073) |
| Osteoarthritis | 0.0634073 | 1.067 (1.041 to 1.094) |
| Osteoporosis | 0.1276254 | 1.135 (1.1 to 1.172) |
| Palliative care | -0.2353552 | 0.774 (0.701 to 0.855) |
| Parkinsonism and tremor | 0.2312839 | 1.266 (1.205 to 1.331) |
| Peptic ulcer disease | 0.0056687 | 1.023 (0.916 to 1.143) |
| Peripheral neuropathy | 0.0335789 | 1.038 (0.994 to 1.084) |
| Peripheral vascular disease | 0.0173065 | 1.021 (0.981 to 1.062) |
| Requirement for care | -0.2177301 | 0.792 (0.75 to 0.836) |
| Respiratory disease | 0.0132757 | 1.018 (0.978 to 1.059) |
| Seizures | 0.2571899 | 1.303 (1.227 to 1.385) |
| Self-harm | 0.1461241 | 1.182 (0.993 to 1.406) |
| Severe mental illness | 0.0676153 | 1.071 (1.026 to 1.119) |
| Skin ulcer | 0.0746926 | 1.08 (1.046 to 1.115) |
| Sleep problems | 0.0056967 | 1.012 (0.961 to 1.066) |
| Social vulnerability | 0.0495075 | 1.061 (1.004 to 1.122) |
| Stress | -0.0200494 | 0.965 (0.9 to 1.035) |
| Stroke | 0.0788542 | 1.086 (1.047 to 1.126) |
| Thyroid problems | -0.0273864 | 0.965 (0.938 to 0.993) |
| Urinary incontinence | 0.0345173 | 1.039 (0.998 to 1.081) |
| Urinary system disease | 0.0119309 | 1.016 (0.988 to 1.044) |
| Visual impairment | 0.02332 | 1.024 (0.996 to 1.053) |
| Washing and bathing | -0.1118824 | 0.853 (0.555 to 1.313) |
| Weakness | -0.0589464 | 0.903 (0.699 to 1.167) |
| Weight loss | 0.0301414 | 1.033 (0.995 to 1.072) |
|  |  |  |
| Constant | -5.954459 | 0.003 (0.002 to 0.003) |

#### Box S3.1: Example calculation for predicting an individual’s risk of falls/fractures within 12 months, using the eFalls model

| **Demonstration of model equation for predicting risk of falls/fractures within 12 months** |
| --- |
| Probability of falls/fractures within 12 months = $\frac{exp(LP)}{1+exp(LP)}$  Where LP = -6.258 + 0.042 x (Age) + 0.330 x (ln((Polypharmacy+1)/10)) – 0.304 (if male)  + 0.490 (if underweight) + 0.240 (if normal weight) – 0.041 (if obese) – 0.145 (if BMI category missing)  + 0.068 (if current smoker) + 0.416 (if harmful drinking) + 0.155 (if higher risk drinking)  + 0.085 (if previous higher risk/harmful drinking) + 0.007 (if zero alcohol)  – 0.068 (if alcohol consumption missing) – 0.064 (if experiencing abdominal pain)  + 0.048 (if experiencing activity limitation) + 0.173 (if experiencing anaemia or haematinic deficiency)  + … [see table S3.1 for values]  And:   - $exp$ is the exponential function - $ln$ is the natural logarithm - Age is measured in years, and polypharmacy is a count of unique drugs* prescribed over the 120 days prior to index date (excluding non-drug chapters of the BNF e.g., bandages), weight groups are defined as underweight if BMI < 18.5; normal weight if 18.5 ≤ BMI < 24.9; obese if BMI ≥ 40. |
| Example: |
| Patient X is an underweight female, aged 89 years, with a history of dementia, liver problems and osteoporosis, who has been prescribed 8 different medications in the last 120 days. She does not smoke and has had previous higher risk/harmful alcohol consumption.  LP = -6.258 + 0.042(89) + 0.330(ln((8+1)/10)) + 0.490 + 0.085 + 0.104 + 0.380 + 0.128  = -1.368  Probability of falls/fractures within 12 months = $\frac{exp(-1.368)}{1+exp(-1.368)}$ = 0.203  Thus, Patient X has a 20.3% risk of falling in the next 12 months. |

* Unique BNF sub-sub-chapters. Combinations of >1 drug from a sub-sub-chapter only counted once towards the total.

### Figure S3.2: Prediction distributions for eFalls model in the model development data.

### Figure S3.3: overlaid normal density plots for the linear predictor distribution of each bootstrap model when applied within that bootstrap sample (navy) and when applied back in the original data)


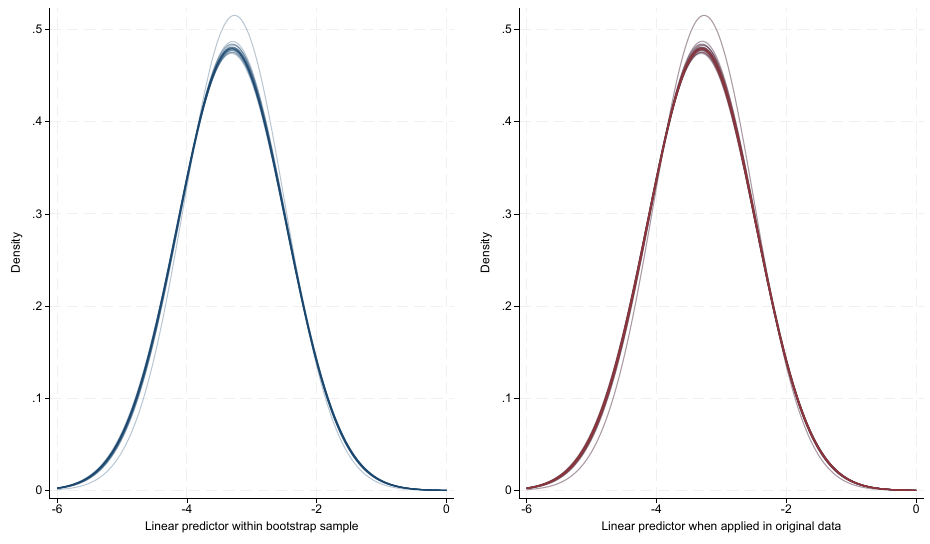


#### Figure S3.4: calibration instability plot, showing lowess smoothed calibration curves for each bootstrap model when applied within that bootstrap sample


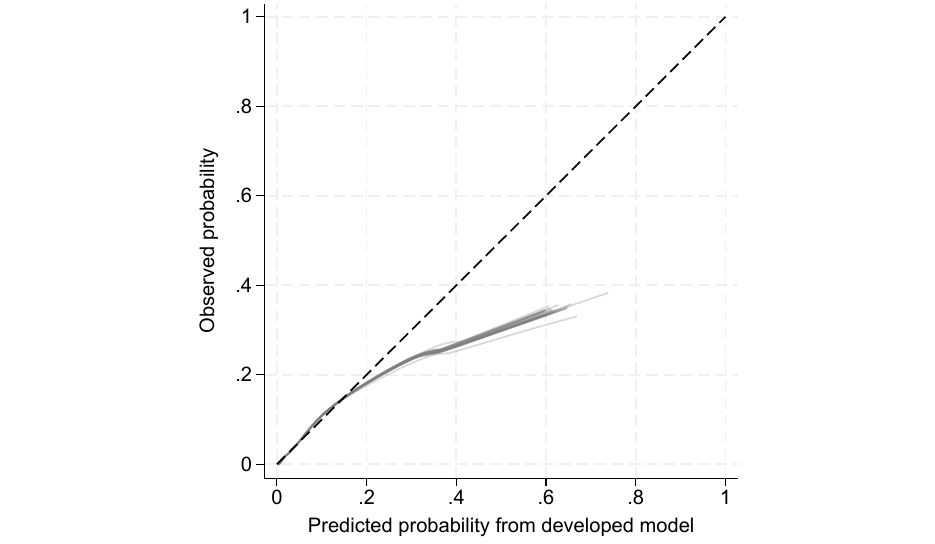


### Table S3.3: Model performance on internal validation: apparent performance of the eFalls prediction model across all practices (no accounting for clustering by GP practice); average optimism in model performance estimates, summarised across bootstrap samples; and optimism-adjusted performance estimates

|  | Apparent performance estimate | Average optimism | Optimism-adjusted estimate |
| --- | --- | --- | --- |
| Calibration slope |  |  |  |
| Point estimate | 1.0083 | 0.0026419 | 1.0057 |
| 95% confidence interval | 0.9953933 to 1.021224 | 0.0002988 to 0.004985 | - |
|  |  |  |  |
| CITL |  |  |  |
| Point estimate | 0.0000 | 0.000277 | -0.0003 |
| 95% confidence interval | -0.0115 to 0.115 | -0.0020746 to 0.0026285 | - |
|  |  |  |  |
| O/E ratio |  |  |  |
| Point estimate | 1.0000 | 0.0002313 | 0.9998 |
| 95% confidence interval | 0.9897182 to 1.010282 | -0.0018962 to 0.0023587 | - |
|  |  |  |  |
| C-statistic |  |  |  |
| Point estimate | 0.7434 | 0.0004245 | 0.7430 |
| 95% confidence interval | 0.74077 to 0.74612 | -0.0000605 to 0.0009096 | - |

### Figure S3.5: Variability in calibration across GP practices (with a minimum of 10 events), showing the variation in calibration performance of the eFalls model when applied across different populations within the development data. Curves across the whole population (without accounting for clustering by practice) are displayed in blue. The dashed line indicates ideal calibration.


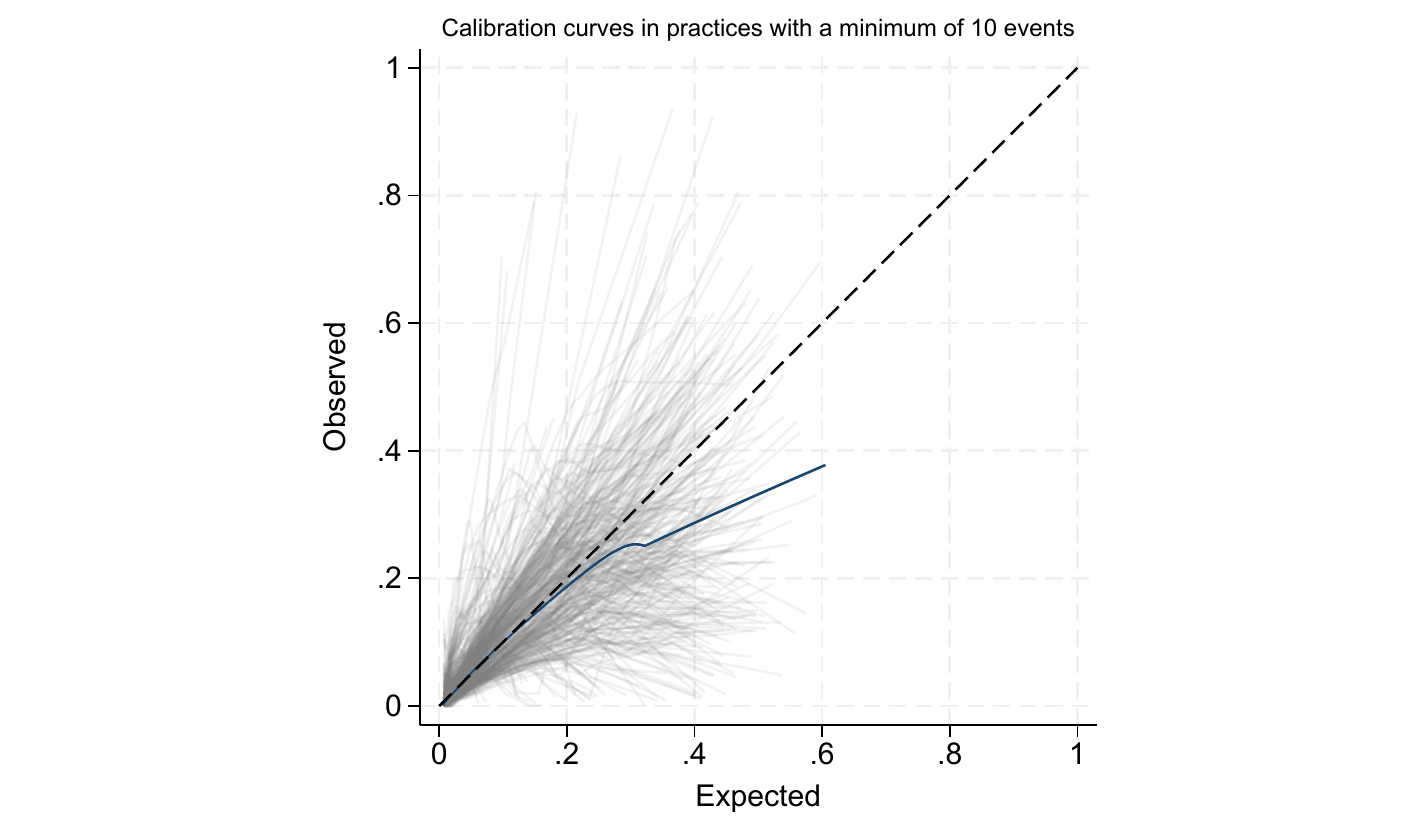


### Figure S3.6: Variability in decision curves across GP practices (with a minimum of 10 events), showing the variation in clinical utility of the eFalls model when applied across different populations within the development data. The decision curve across the whole population (without accounting for clustering by practice) is displayed in blue. The solid black line indicates the net benefit of a “treat all” strategy across the whole population, while the horizontal line shows the net benefit of a “treat none” strategy.


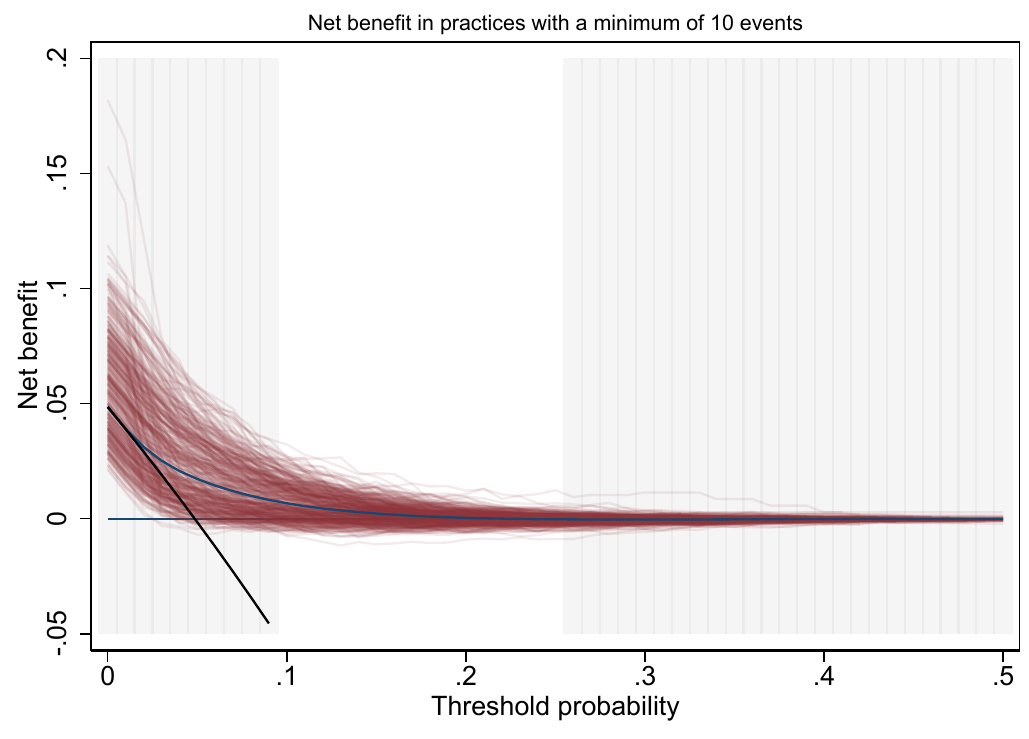


### Figure S3.7: Variability in clinical utility across curves across GP practices (with a minimum of 10 events), showing the median difference between the standardised net benefit (SNB) of the mortality model and alternative strategies (treat all, treat none, and “next best” of the two) in that sub-population. The median difference is shown across a range of potential threshold probability values (from 0 to 0.5), with bands showing where 50%, 80% and 90% of GP practices lie. The red horizontal line shows the point where using the model is no better than the next best alternative. The green vertical line shows where the next best alternative strategy changes (at a threshold probability of 0.06, or 6%).

| 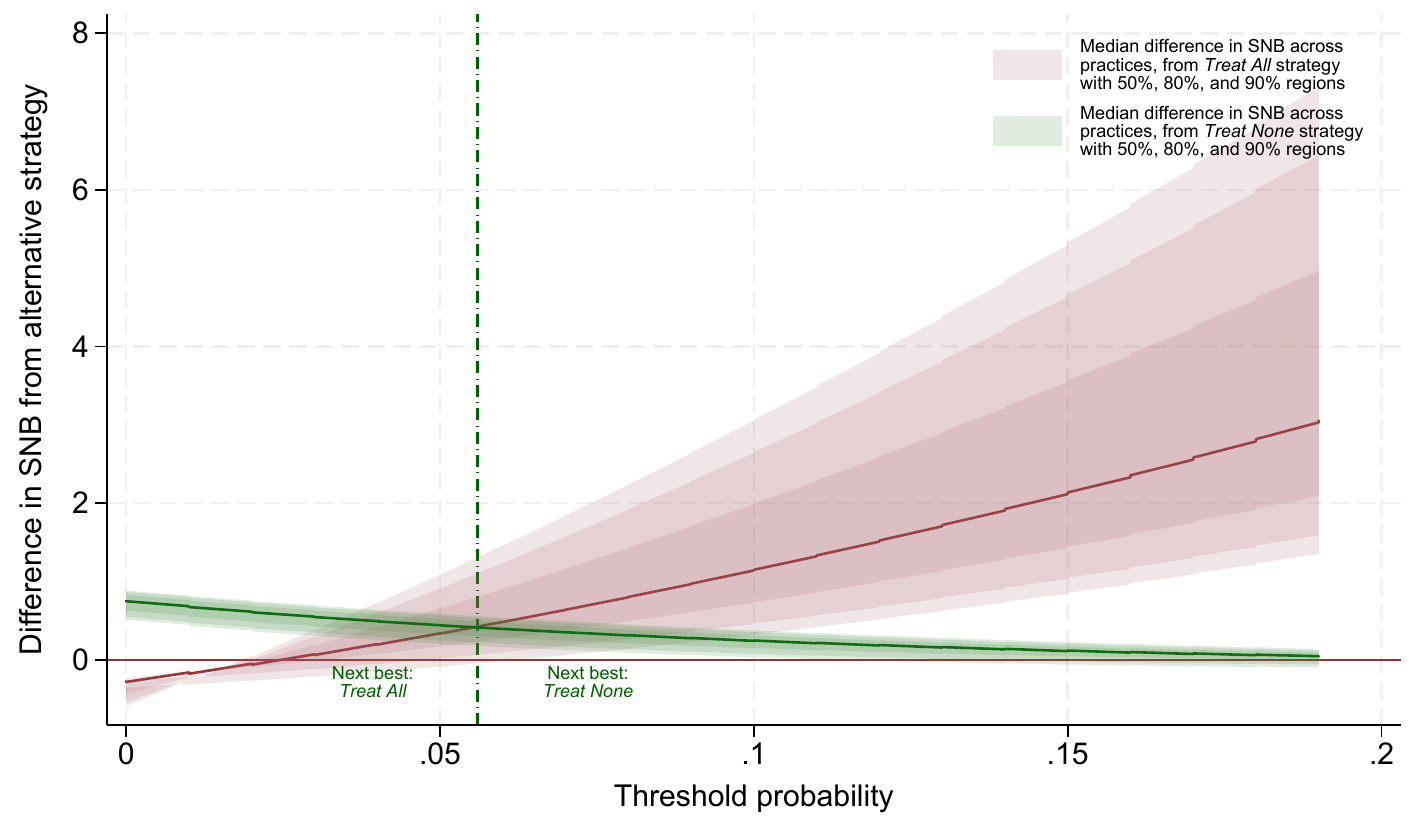 |
| --- |
| 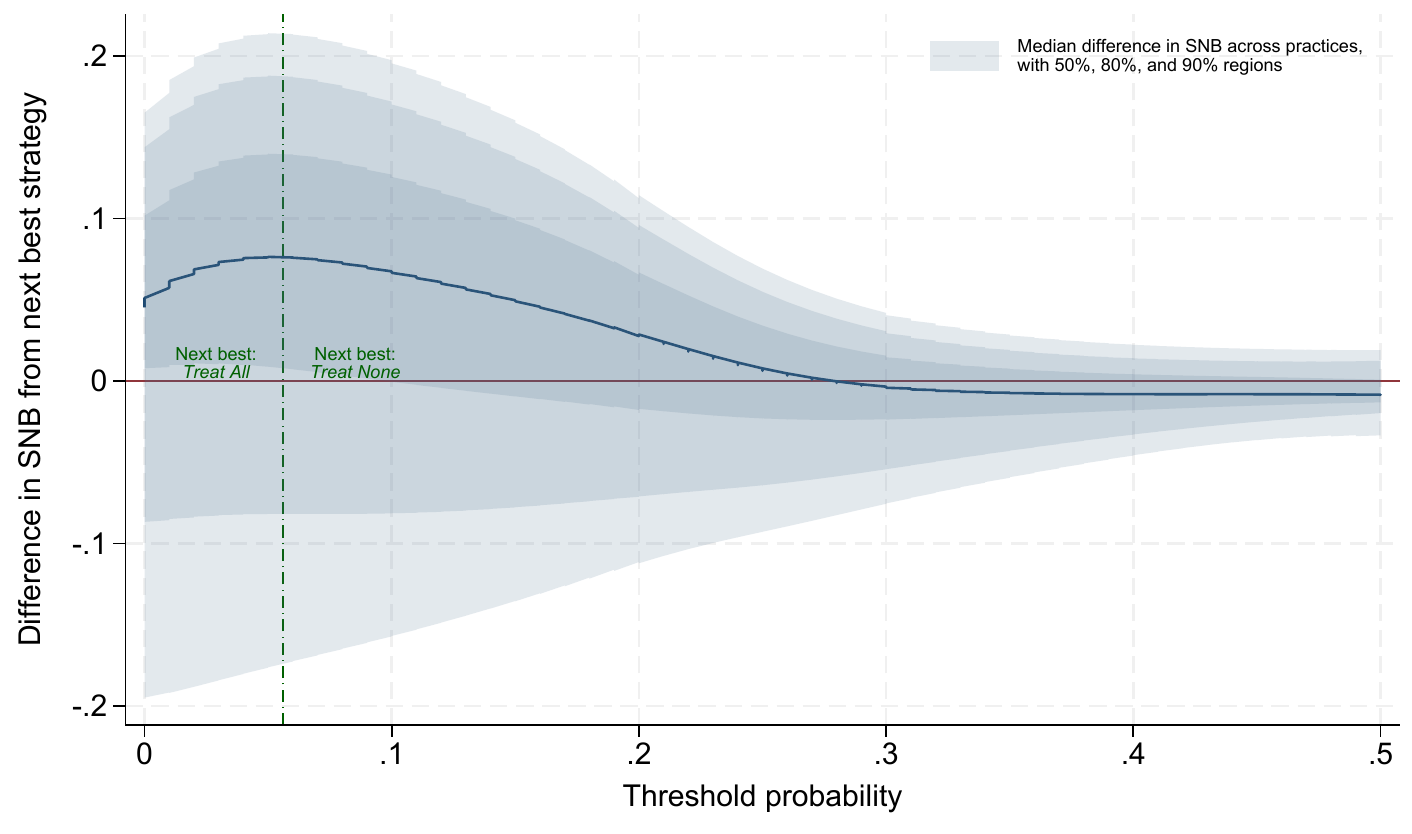 |

## Internal External Cross Validation

#### Figure S3.8: Prediction distributions for fall/fracture model. Each plot shows the distribution of predictions from the model generated in the data excluding a particular WIMD 2019 subgroup, when the model was applied ‘externally’ in that omitted group. Summaries of linear predictor distributions in each WIMD group are provided.


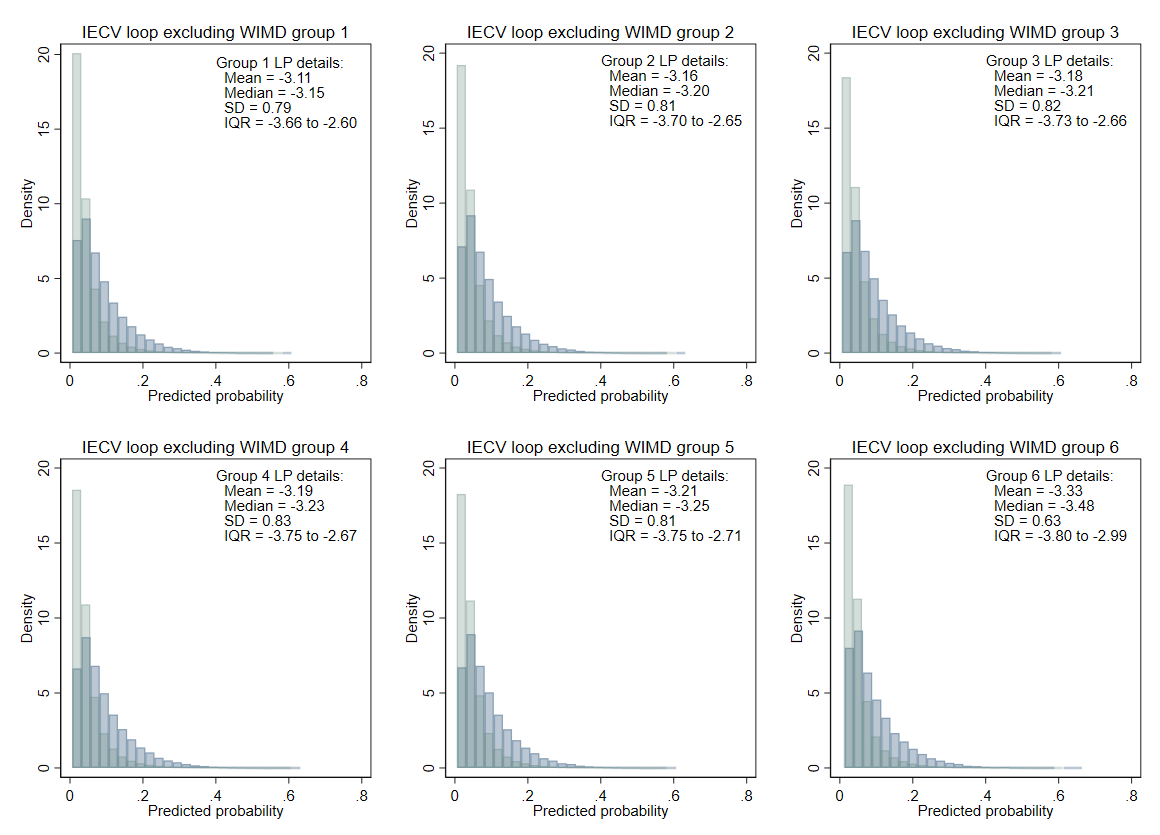


#### Figure S3.9: Calibration plots for models developed across IECV cycles. Each plot shows the calibration of the model generated in the data excluding a particular WIMD 2019 subgroup, when the model was applied ‘externally’ in that omitted group. Summaries of model performance in each WIMD group are provided.


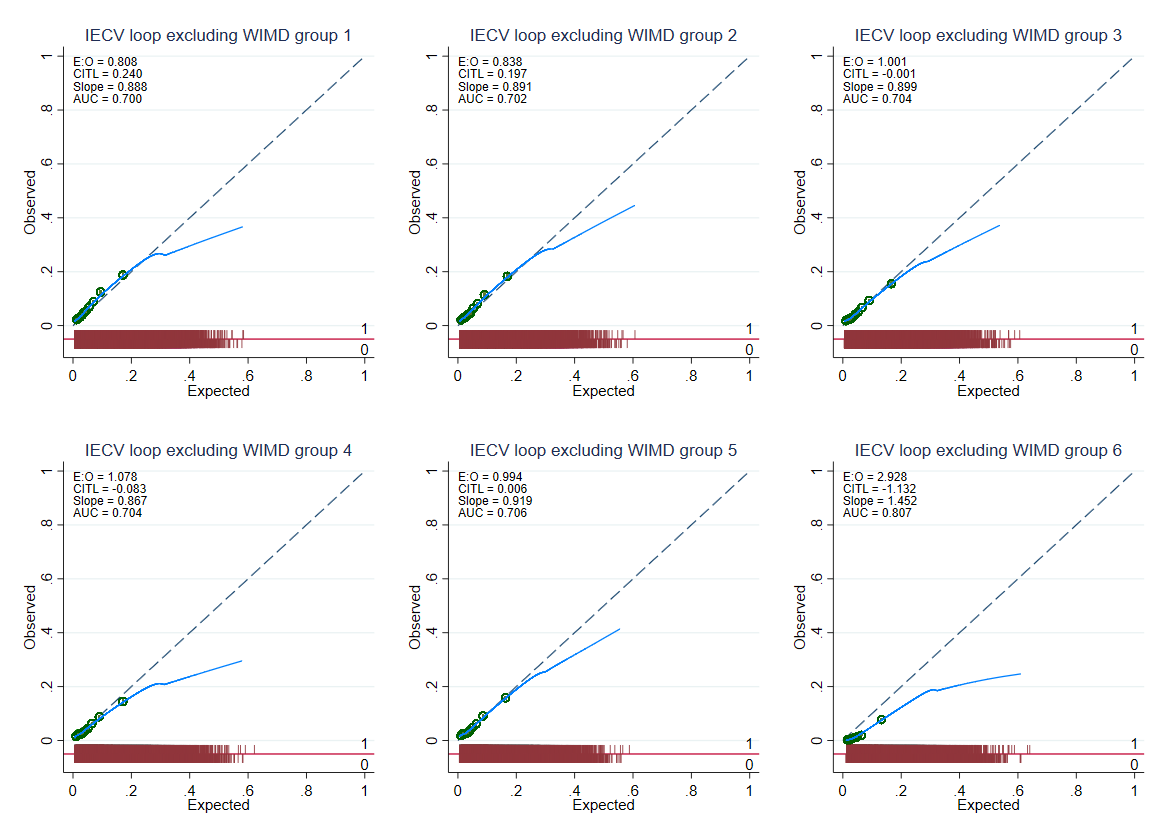


#### Figure S3.10: Forest plots of model performance statistics for models developed across IECV cycles. Statistics refer to the performance of the model generated in the data excluding a particular WIMD 2019 subgroup, when the model was applied ‘externally’ in that omitted group. Pooled estimates summarise model performance across all IECV cycles


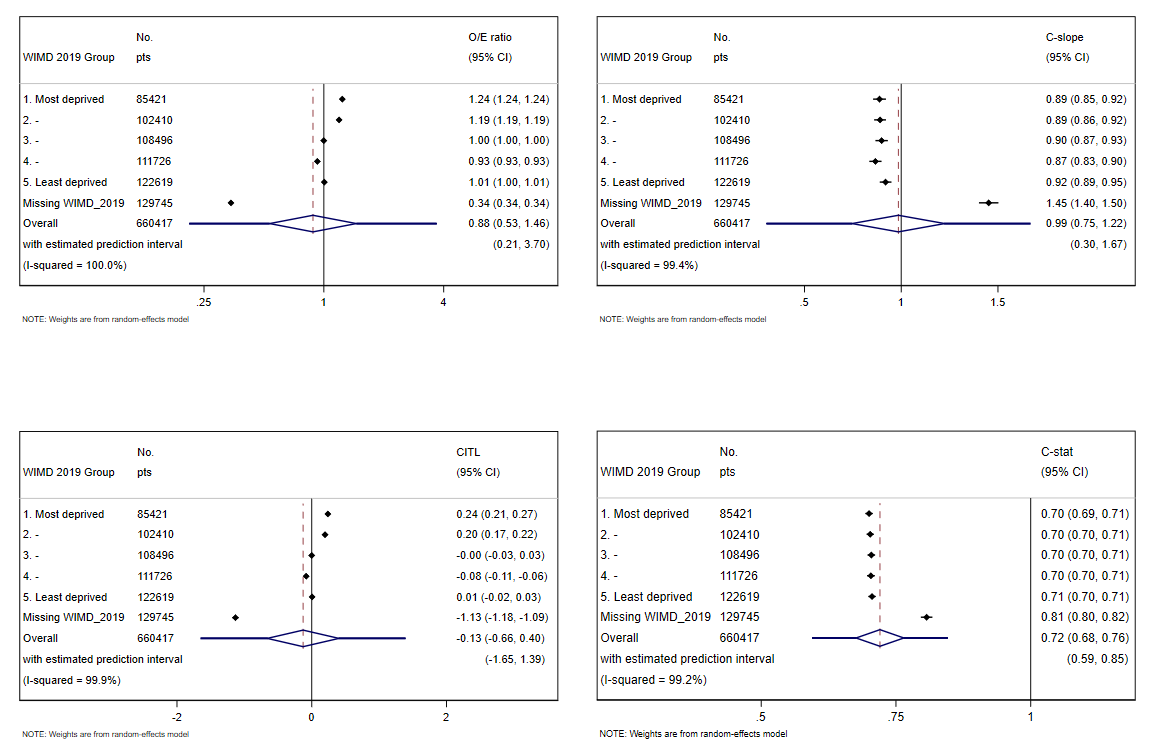


## External validation

### Box S3.2: Example calculation for predicting an individual’s risk of falls/fractures within 12 months, using the recalibrated version of the eFalls model

| **Demonstration of recalibrated model equation for predicting risk of falls/fractures within 12 months** |
| --- |
| Probability of falls/fractures within 12 months = $\frac{exp(-0.423+1.25*LP)}{1+exp(-0.423+1.25*LP)}$  Where LP = -6.258 + 0.042 x (Age) + 0.330 x (ln((Polypharmacy+1)/10)) – 0.304 (if male)  + 0.490 (if underweight) + 0.240 (if normal weight) – 0.041 (if obese) – 0.145 (if BMI category missing)  + 0.068 (if current smoker) + 0.416 (if harmful drinking) + 0.155 (if higher risk drinking)  + 0.085 (if previous higher risk/harmful drinking) + 0.007 (if zero alcohol)  – 0.068 (if alcohol consumption missing) – 0.064 (if experiencing abdominal pain)  + 0.048 (if experiencing activity limitation) + 0.173 (if experiencing anaemia or haematinic deficiency)  + … [see table S3.1 for values]  And:   - $exp$ is the exponential function - $ln$ is the natural logarithm - Age is measured in years, and polypharmacy is a count of unique drugs* prescribed over the 120 days prior to index date (excluding non-drug chapters of the BNF e.g., bandages), weight groups are defined as underweight if BMI < 18.5; normal weight if 18.5 ≤ BMI < 24.9; obese if BMI ≥ 40. |
| Example: |
| Patient X is an underweight female, aged 89 years, with a history of dementia, liver problems and osteoporosis, who has been prescribed 8 different medications in the last 120 days. She does not smoke and has had previous higher risk/harmful alcohol consumption.  LP = -6.258 + 0.042(89) + 0.330(ln((8+1)/10)) + 0.490 + 0.085 + 0.104 + 0.380 + 0.128  = -1.368  Probability of falls/fractures within 12 months = $\frac{exp(-0.423+1.25*(-1.368))}{1+exp(-0.423+1.25*(-1.368))}$ = 0.106  Thus, Patient X has a 10.6% risk of falling in the next 12 months, from the recalibrated model. |

* Unique BNF sub-sub-chapters. Combinations of >1 drug from a sub-sub-chapter only counted once towards the total.

### Figure S3.11: Prediction distributions for eFalls model, before and after recalibration, in the external validation data.

| External validation | Apparent validation after recalibration |
| --- | --- |
| 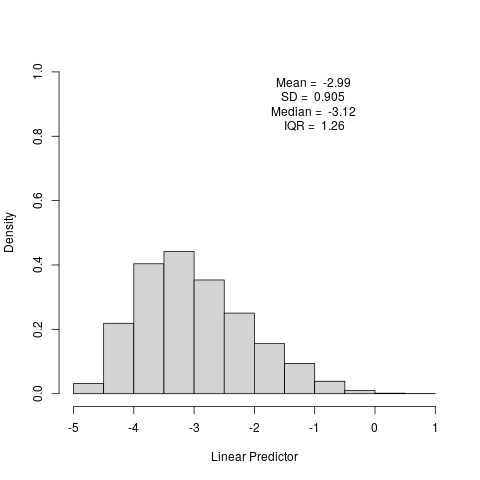 | 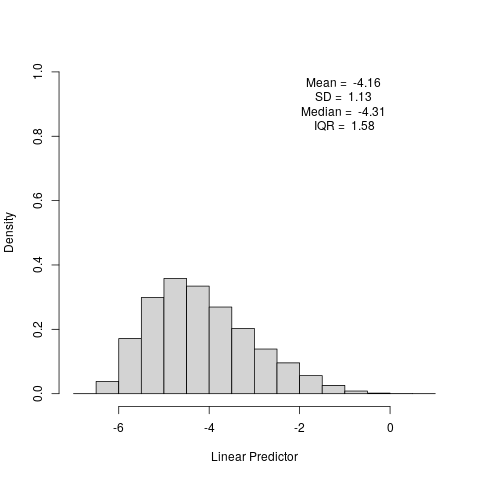 |

### Figure S3.12: Variability in calibration across GP practices, showing the variation in calibration performance of the eFalls model, before and after recalibration, when applied across different populations within the external validation data. Curves across the whole population (without accounting for clustering by practice) are displayed in blue. The dashed line indicates ideal calibration.

| External validation | Apparent validation after recalibration |
| --- | --- |
| 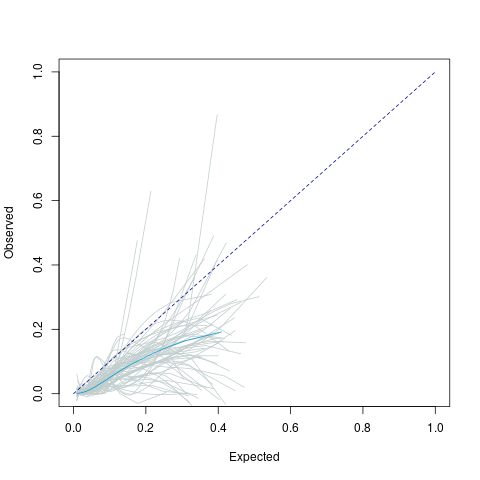 | 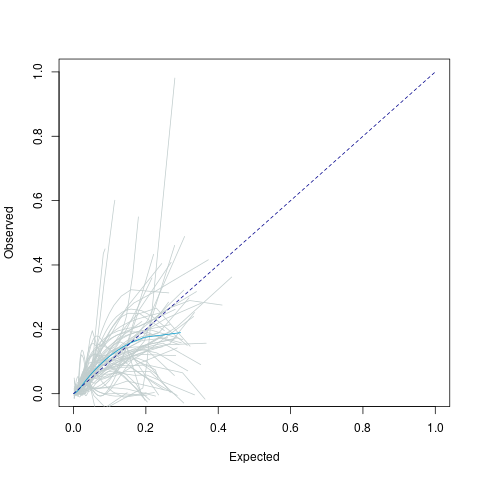 |

### Figure S3.13: Variability in decision curves across GP practices, showing the variation in clinical utility of the eFalls model, before and after recalibration, when applied across different populations within the development data. The decision curve across the whole population (without accounting for clustering by practice) is displayed as a solid blue line. The solid black line indicates the net benefit of a “treat all” strategy across the whole population, while the horizontal line shows the net benefit of a “treat none” strategy.

| External validation | Apparent validation after recalibration |
| --- | --- |
| 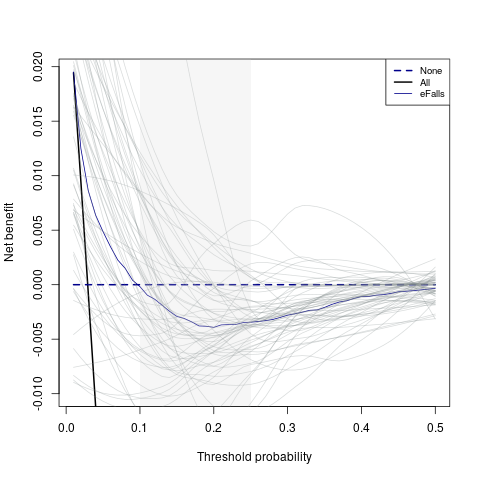 | 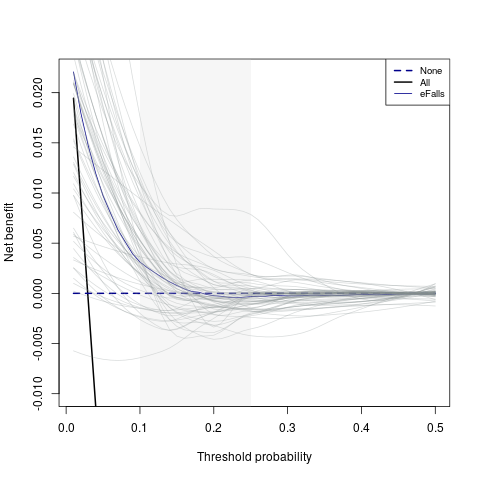 |

### Figure S3.14: Variability in performance of the recalibrated prediction model on apparent validation across GP practices. Plots show calibration slope, calibration-in-the-large, observed/expected ratio, and c-statistic plotted against their standard error within each practice. Bounds show 95% prediction intervals for the performance measure across possible standard errors.

| 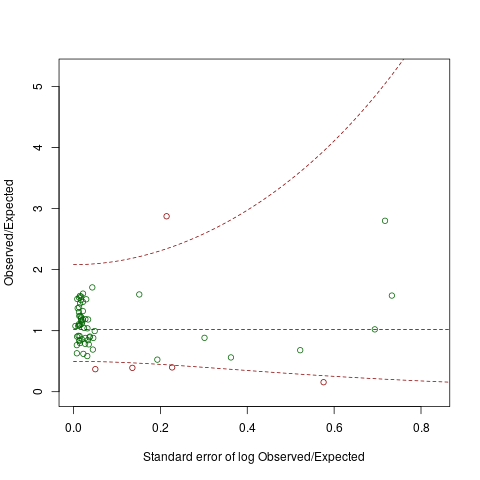 | 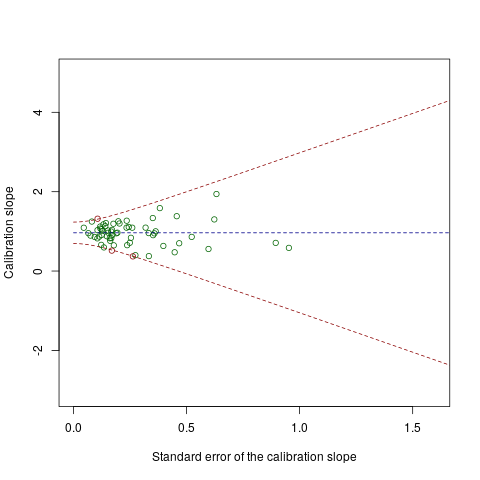 |
| --- | --- |
| 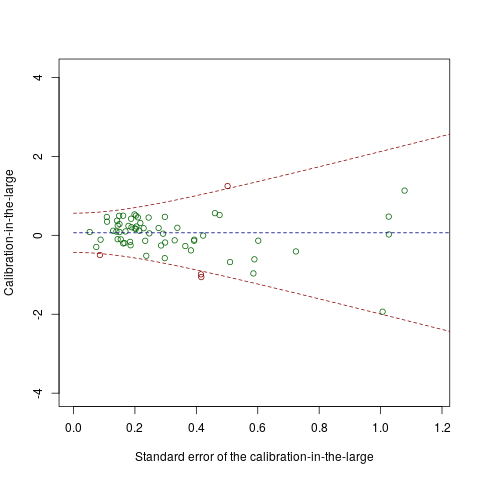 | 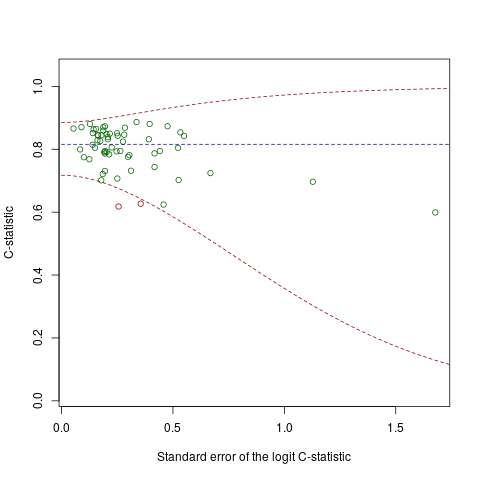 |

### Table S3.4: Performance statistics on external validation of the eFalls model, before recalibration, with 95% confidence intervals, calculated in clinically relevant subgroups

|  |  | Calibration | | | Discrimination |
| --- | --- | --- | --- | --- | --- |
| Subgroup | n | O/E ratio | Calibration slope | CITL | C-statistic |
| IMD |  |  |  |  |  |
| 1. Most deprived | 18,600 | 0.454 (0.448 to 0.466) | 1.123 (1.126 to 1.158) | -0.885 (-0.899 to -0.857) | 0.788 (0.789 to 0.796) |
| 2. - | 12,162 | 0.436 (0.426 to 0.450) | 1.106 (1.078 to 1.116) | -0.924 (-0.949 to -0.892) | 0.793 (0.788 to 0.796) |
| 3. - | 14,337 | 0.488 (0.485 to 0.500) | 1.338 (1.308 to 1.341) | -0.804 (-0.811 to -0.777) | 0.849 (0.844 to 0.849) |
| 4. - | 11,172 | 0.390 (0.381 to 0.402) | 1.413 (1.382 to 1.433) | -1.036 (-1.063 to -1.006) | 0.867 (0.862 to 0.871) |
| 5. - Least deprived | 7,582 | 0.460 (0.447 to 0.470) | 1.307 (1.277 to 1.334) | -0.857 (-0.890 to -0.833) | 0.844 (0.841 to 0.851) |
| Missing IMD | 17,832 | 0.373 (0.365 to 0.376) | 1.304 (1.265 to 1.325) | -1.086 (-1.112 to -1.079) | 0.835 (0.827 to 0.837) |
|  |  |  |  |  |  |
| eFI frailty group |  |  |  |  |  |
| Fit | 32,732 | 0.201 (0.192 to 0.206) | 1.558 (1.504 to 1.590) | -1.636 (-1.683 to -1.613) | 0.749 (0.744 to 0.758) |
| Mild frailty | 24,694 | 0.313 (0.311 to 0.324) | 1.300 (1.234 to 1.298) | -1.221 (-1.230 to -1.187) | 0.735 (0.722 to 0.733) |
| Moderate frailty | 14,496 | 0.514 (0.510 to 0.526) | 1.083 (1.060 to 1.112) | -0.744 (-0.753 to -0.719) | 0.703 (0.699 to 0.707) |
| Severe frailty | 9,763 | 0.583 (0.577 to 0.593) | 0.749 (0.721 to 0.764) | -0.665 (-0.678 to -0.647) | 0.643 (0.638 to 0.646) |
|  |  |  |  |  |  |
| Sex |  |  |  |  |  |
| Female | 44,366 | 0.417 (0.413 to 0.420) | 1.247 (1.230 to 1.262) | -0.986 (-0.997 to -0.978) | 0.818 (0.815 to 0.821) |
| Male | 37,319 | 0.463 (0.462 to 0.476) | 1.368 (1.371 to 1.393) | -0.832 (-0.834 to -0.803) | 0.834 (0.835 to 0.839) |
|  |  |  |  |  |  |
| BMI group |  |  |  |  |  |
| Underweight | 1,742 | 0.452 (0.444 to 0.492) | 0.845 (0.842 to 0.924) | -0.970 (-1.000 to -0.875) | 0.717 (0.715 to 0.733) |
| Normal | 24,862 | 0.433 (0.426 to 0.439) | 1.248 (1.231 to 1.265) | -0.948 (-0.965 to -0.932) | 0.823 (0.821 to 0.826) |
| Overweight | 29,631 | 0.436 (0.433 to 0.448) | 1.336 (1.325 to 1.362) | -0.906 (-0.913 to -0.877) | 0.828 (0.828 to 0.834) |
| Obese | 21,698 | 0.380 (0.379 to 0.392) | 1.389 (1.366 to 1.426) | -1.050 (-1.054 to -1.018) | 0.831 (0.826 to 0.836) |
| Missing BMI | 3,752 | 0.797 (0.763 to 0.835) | 1.246 (1.234 to 1.336) | -0.248 (-0.299 to -0.200) | 0.819 (0.815 to 0.831) |

### Figure S3.15: Calibration plots and decision curves for external validation of the eFalls model, before recalibration, in subgroups by IMD quintile

| IMD group 1 | 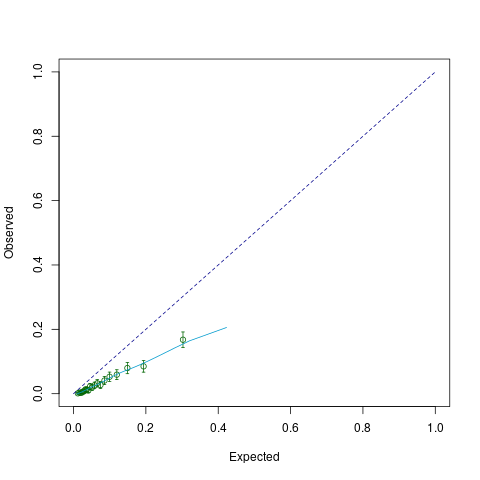 | 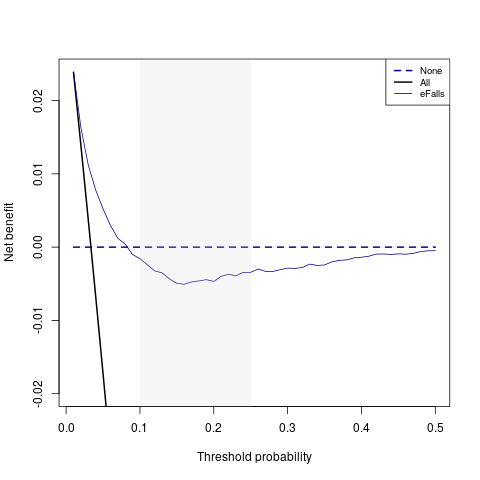 |
| --- | --- | --- |
| IMD group 2 | 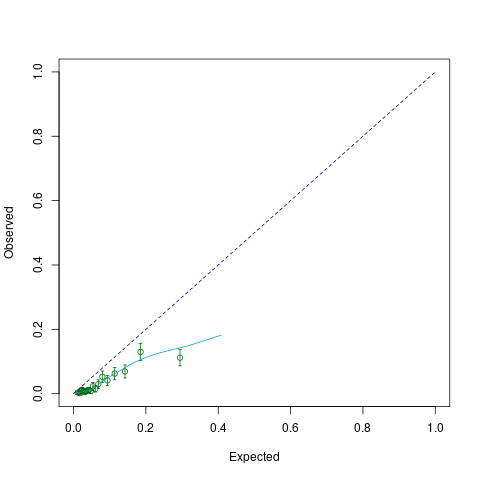 | 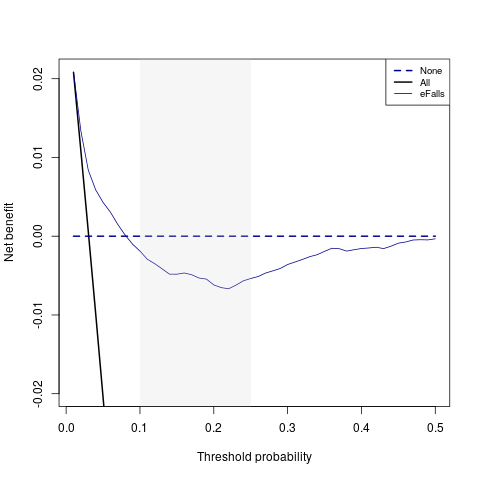 |
| IMD group 3 | 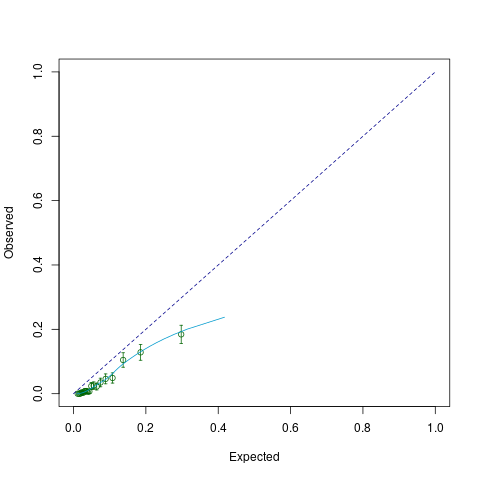 | 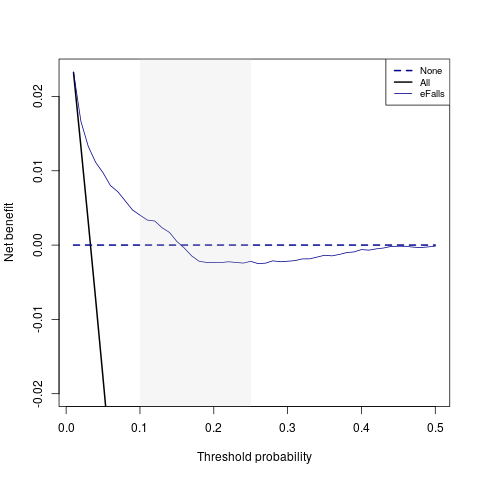 |
| IMD group 4 | 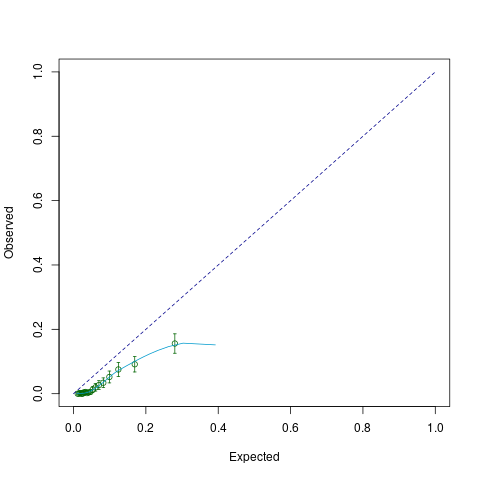 | 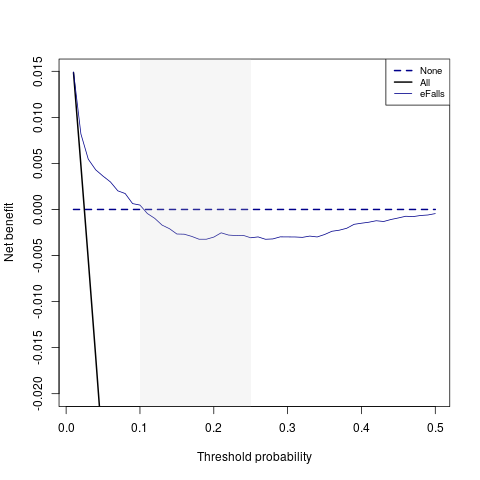 |
| IMD group 5 | 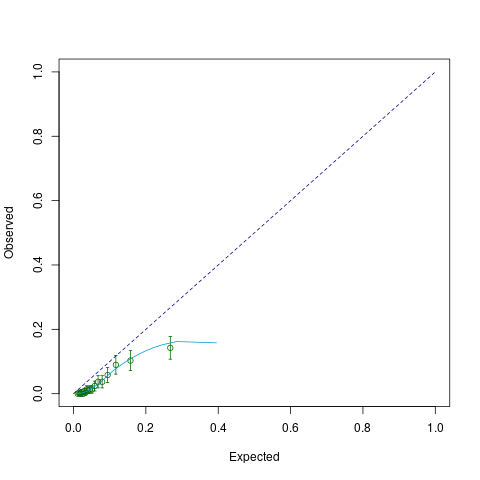 | 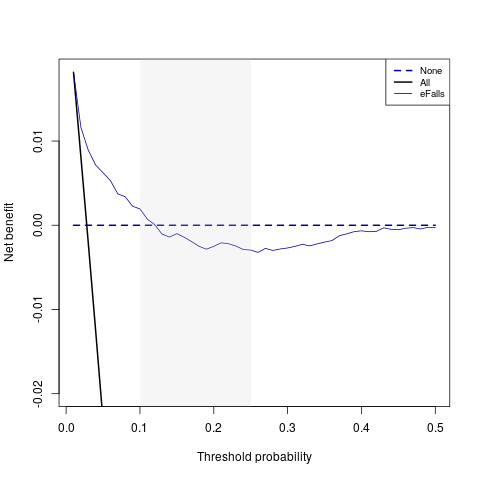 |
| Group missing IMD | 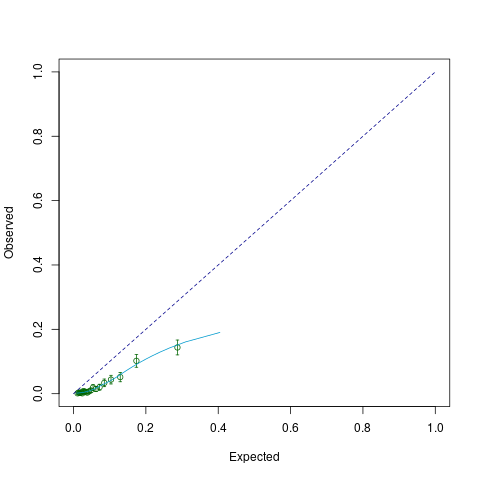 | 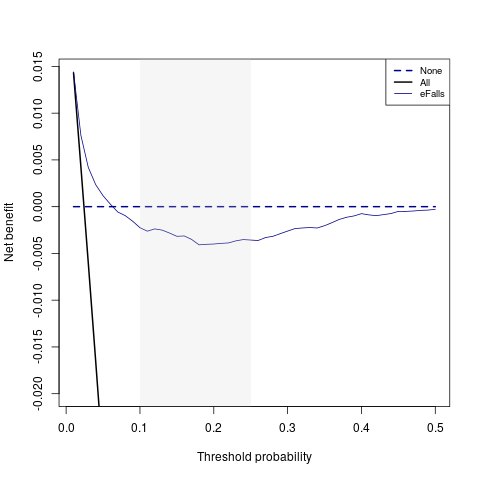 |

### Figure S3.16: Calibration plots and decision curves for external validation of the eFalls model, before recalibration, in frailty subgroups,

| Fit subgroup | 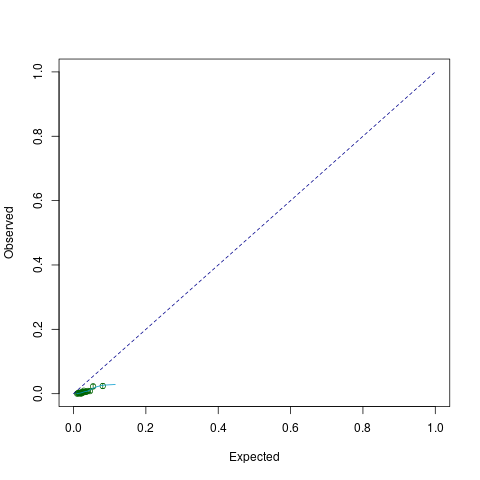 | 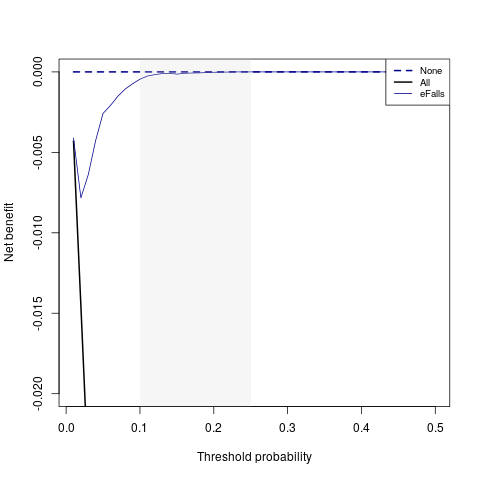 |
| --- | --- | --- |
| Mild frailty subgroup | 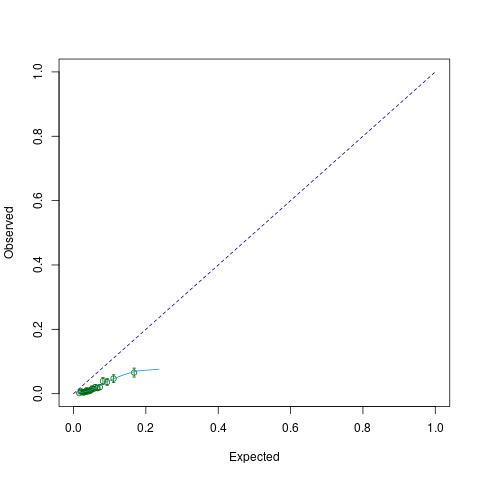 | 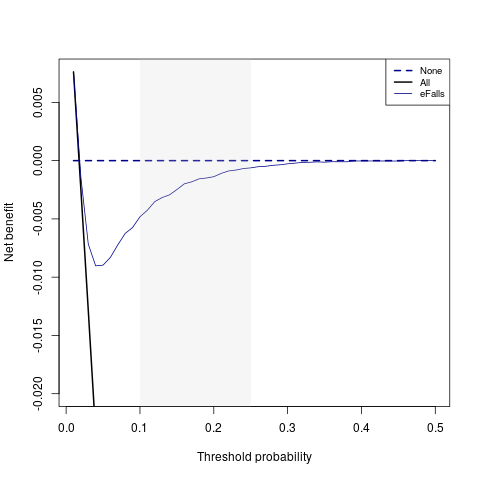 |
| Moderate frailty subgroup | 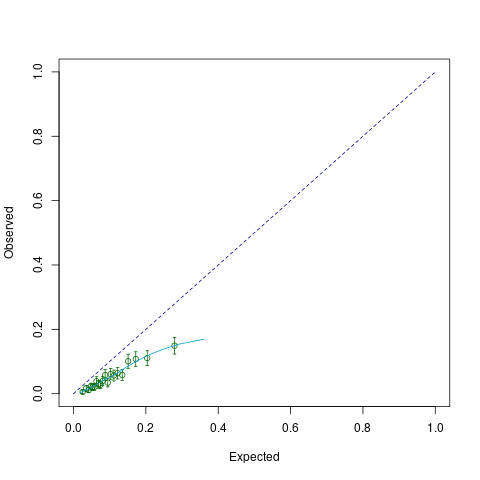 | 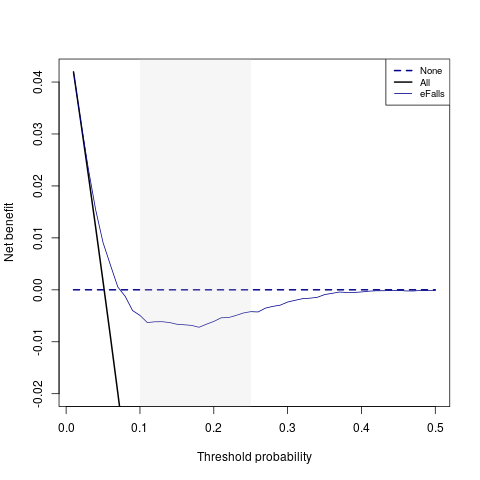 |
| Severe frailty subgroup | 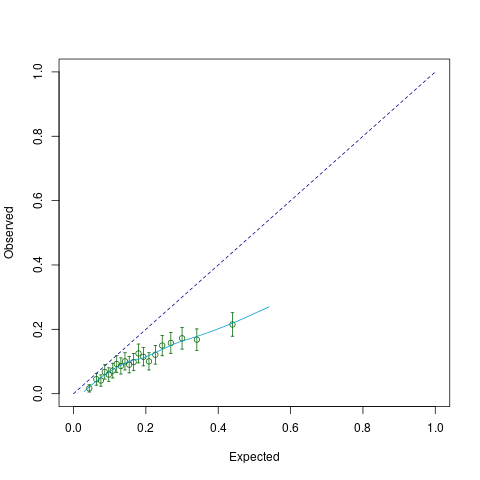 | 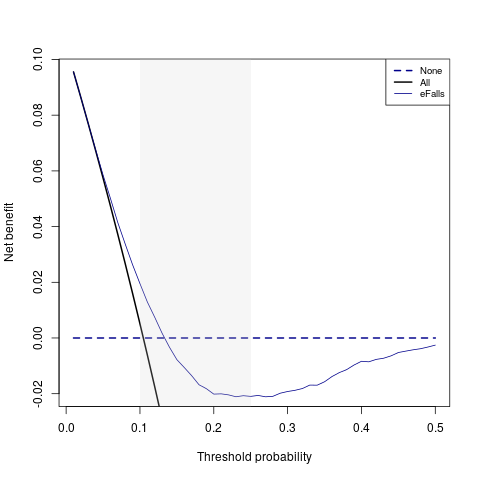 |

### Figure S3.17: Calibration plots and decision curves for external validation of the eFalls model, before recalibration, in sex subgroups

| Female subgroup | 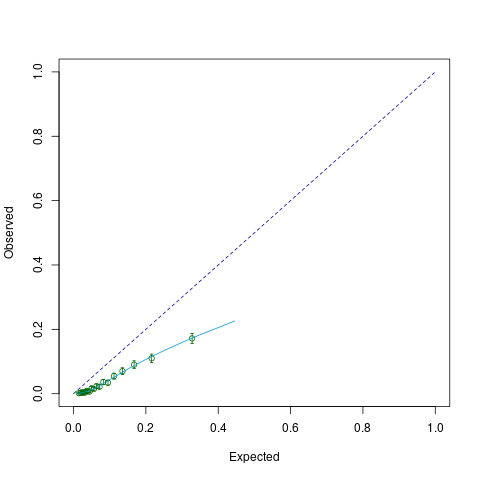 | 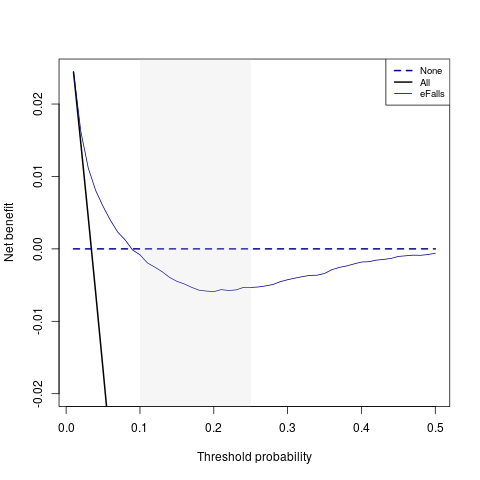 |
| --- | --- | --- |
| Male subgroup | 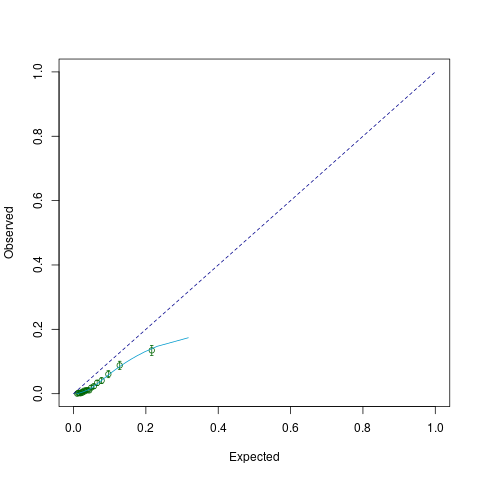 | 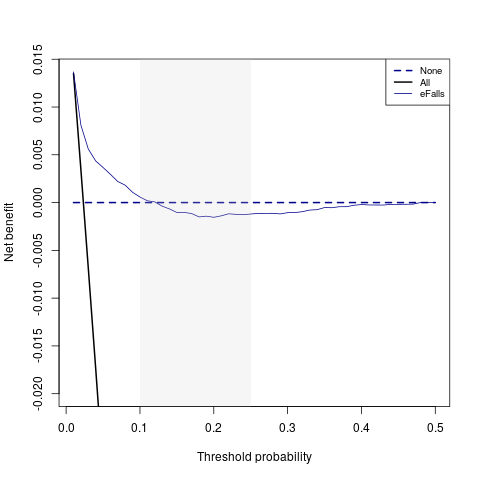 |

### Figure S3.18: Calibration plots and decision curves for external validation of the eFalls model, before recalibration, in BMI subgroups

| Underweight BMI subgroup | 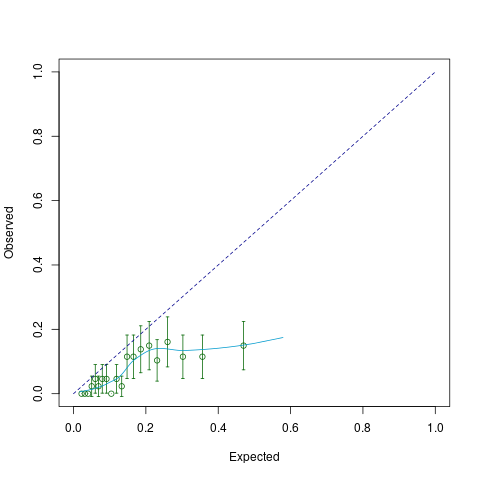 | 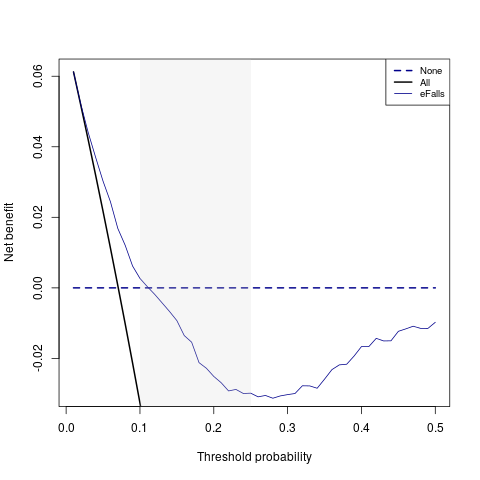 |
| --- | --- | --- |
| Normal weight BMI subgroup | 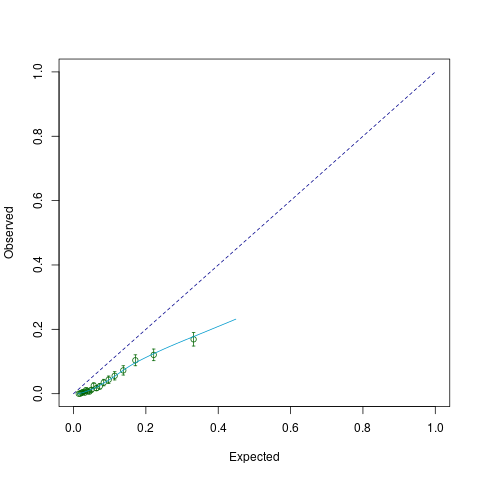 | 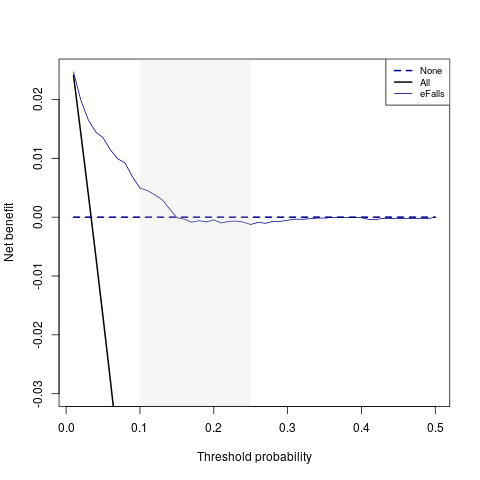 |
| Overweight BMI subgroup | 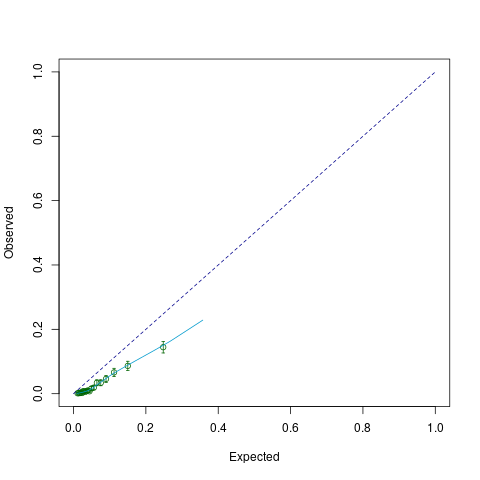 | 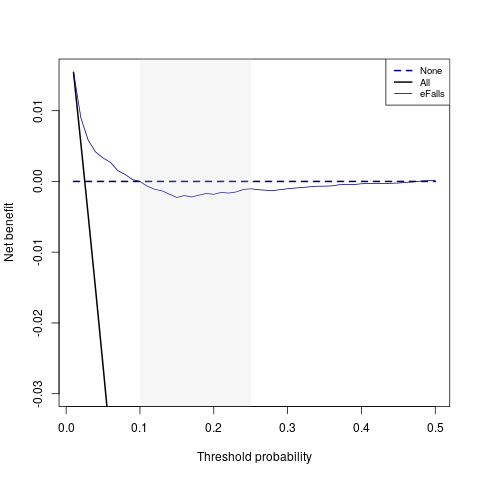 |
| Obese BMI subgroup | 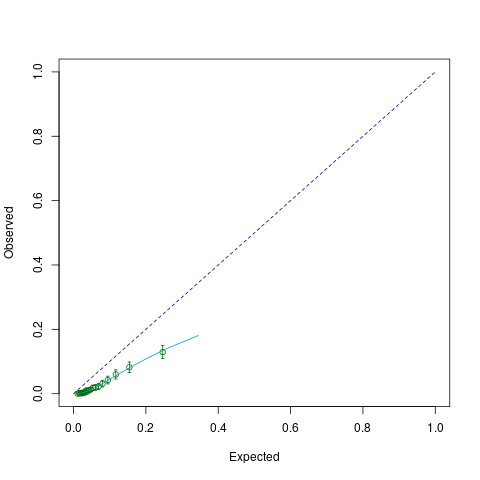 | 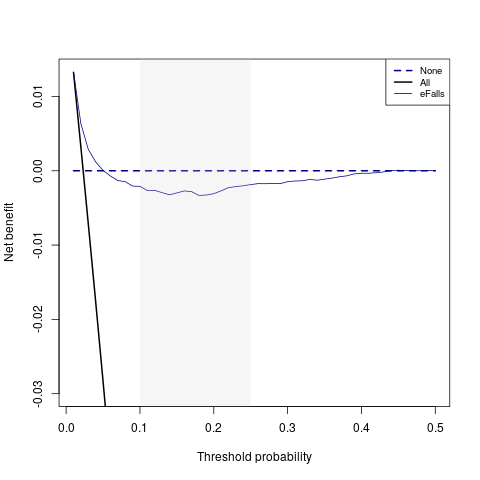 |
| Missing BMI subgroup | 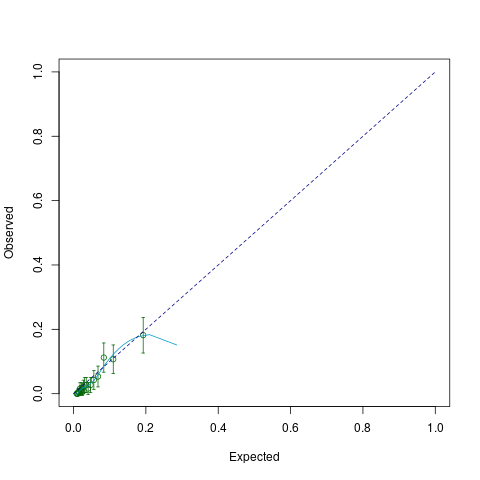 | 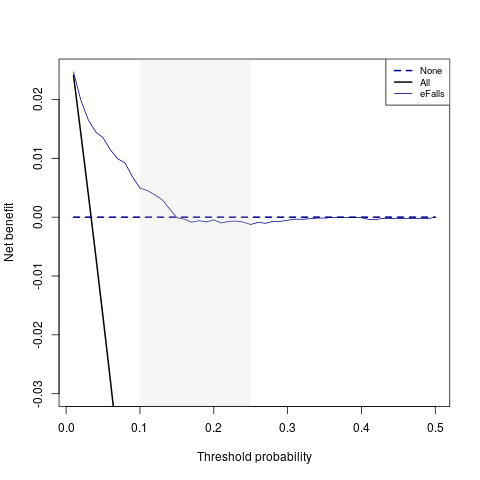 |

### Table S3.4: Expected number of True Positives (TP), False Positives (FP), True Negatives (TN) and False Negatives (FN) per 1000 older adults assessed using the eFalls model (before recalibration) with different predicted probability thresholds to assign “high risk”. Numbers are based on the eFalls model’s performance on external validation in the Connected Bradford data.

| Threshold probability | TP per 1000 | FP per 1000 | TN per 1000 | FN per 1000 | Sensitivity | Specificity |
| --- | --- | --- | --- | --- | --- | --- |
| 0.10 | 19.2 | 174.3 | 796.5 | 10.1 | 0.66 | 0.82 |
| 0.11 | 17.8 | 151.9 | 818.8 | 11.4 | 0.61 | 0.84 |
| 0.12 | 16.6 | 131.9 | 838.9 | 12.6 | 0.57 | 0.86 |
| 0.13 | 15.5 | 116.6 | 854.2 | 13.7 | 0.53 | 0.88 |
| 0.14 | 14.4 | 103.7 | 867.0 | 14.8 | 0.49 | 0.89 |
| 0.15 | 13.2 | 91.5 | 879.3 | 16.0 | 0.45 | 0.91 |
| 0.16 | 12.3 | 80.9 | 889.8 | 16.9 | 0.42 | 0.92 |
| 0.17 | 11.3 | 71.6 | 899.2 | 18.0 | 0.38 | 0.93 |
| 0.18 | 10.3 | 64.0 | 906.8 | 18.9 | 0.35 | 0.93 |
| 0.19 | 9.4 | 56.4 | 914.4 | 19.8 | 0.32 | 0.94 |
| 0.20 | 8.6 | 50.2 | 920.5 | 20.6 | 0.30 | 0.95 |
| 0.21 | 8.0 | 44.0 | 926.8 | 21.2 | 0.27 | 0.95 |
| 0.22 | 7.4 | 39.1 | 931.7 | 21.9 | 0.25 | 0.96 |
| 0.23 | 6.7 | 34.6 | 936.2 | 22.6 | 0.23 | 0.96 |
| 0.24 | 6.1 | 30.2 | 940.6 | 23.2 | 0.21 | 0.97 |
| 0.25 | 5.4 | 26.6 | 944.2 | 23.8 | 0.19 | 0.97 |

### Table S3.5: Expected number of True Positives (TP), False Positives (FP), True Negatives (TN) and False Negatives (FN) per 1000 older adults assessed using the recalibrated model with different predicted probability thresholds to assign “high risk”. Numbers are based on the model’s performance on apparent validation, after recalibration to the Connected Bradford data.

| Threshold probability | TP per 1000 | FP per 1000 | TN per 1000 | FN per 1000 | Sensitivity | Specificity |
| --- | --- | --- | --- | --- | --- | --- |
| 0.10 | 9.1 | 53.6 | 917.2 | 20.2 | 0.31 | 0.95 |
| 0.11 | 8.1 | 45.2 | 925.5 | 21.1 | 0.28 | 0.95 |
| 0.12 | 7.3 | 38.3 | 932.5 | 22.0 | 0.25 | 0.96 |
| 0.13 | 6.5 | 32.7 | 938.1 | 22.8 | 0.22 | 0.97 |
| 0.14 | 5.6 | 27.5 | 943.2 | 23.6 | 0.19 | 0.97 |
| 0.15 | 5.0 | 23.9 | 946.9 | 24.3 | 0.17 | 0.98 |
| 0.16 | 4.3 | 20.5 | 950.3 | 24.9 | 0.15 | 0.98 |
| 0.17 | 3.8 | 17.8 | 952.9 | 25.4 | 0.13 | 0.98 |
| 0.18 | 3.5 | 15.2 | 955.5 | 25.8 | 0.12 | 0.98 |
| 0.19 | 3.0 | 13.2 | 957.5 | 26.3 | 0.10 | 0.99 |
| 0.20 | 2.6 | 11.3 | 959.4 | 26.7 | 0.09 | 0.99 |
| 0.21 | 2.3 | 9.6 | 961.2 | 27.0 | 0.08 | 0.99 |
| 0.22 | 2.0 | 8.4 | 962.4 | 27.3 | 0.07 | 0.99 |
| 0.23 | 1.7 | 7.2 | 963.5 | 27.5 | 0.06 | 0.99 |
| 0.24 | 1.6 | 6.2 | 964.5 | 27.7 | 0.05 | 0.99 |
| 0.25 | 1.4 | 5.3 | 965.5 | 27.8 | 0.05 | 0.99 |

# References

1. Debray TPA, Collins GS, Riley RD, Snell KIE, Van Calster B, Reitsma JB, et al. Transparent reporting of multivariable prediction models developed or validated using clustered data: TRIPOD-Cluster checklist. Bmj. 2023;380:e071018.

2. Riley RD, Snell KI, Ensor J, Burke DL, Harrell FE, Jr., Moons KG, et al. Minimum sample size for developing a multivariable prediction model: PART II - binary and time-to-event outcomes. Stat Med. 2019;38(7):1276-96.

3. Clegg A, Bates C, Young J, Ryan R, Nichols L, Ann Teale E, et al. Development and validation of an electronic frailty index using routine primary care electronic health record data. Age Ageing. 2016;45(3):353-60.

4. Riley RD, Debray TPA, Collins GS, Archer L, Ensor J, van Smeden M, et al. Minimum sample size for external validation of a clinical prediction model with a binary outcome. Statistics in Medicine. 2021;40(19):4230-51.

5. Goldacre B, Morely J. Better, broader, safer: using health data for research and analysis. A review commissioned by the Secretary of state for health and social care. Department of Health and Social Care. 2022.
